# Racial and Ethnic Inequities in the Return-to-Work of Workers Experiencing Injury or Illness: A Systematic Review

**DOI:** 10.1007/s10926-023-10119-1

**Published:** 2023-06-09

**Authors:** Arif Jetha, Lahmea Navaratnerajah, Faraz Vahid Shahidi, Nancy Carnide, Aviroop Biswas, Basak Yanar, Arjumand Siddiqi

**Affiliations:** 1 Institute for Work & Health, Suite 1800, 400 University Avenue, M5G 1S5 Toronto, ON Canada; 2 Dalla Lana School of Public Health, University of Toronto, Toronto, ON Canada

**Keywords:** Racism, Return to work, Racial factors, Ethnic and racial minorities, Back to work

## Abstract

**Purpose:**

Non-White workers face more frequent, severe, and disabling occupational and non-occupational injuries and illnesses when compared to White workers. It is unclear whether the return-to-work (RTW) process following injury or illness differs according to race or ethnicity.

**Objective:**

To determine racial and ethnic differences in the RTW process of workers with an occupational or non-occupational injury or illness.

**Methods:**

A systematic review was conducted. Eight academic databases - Medline, Embase, PsycINFO, CINAHL, Sociological Abstracts, ASSIA, ABI Inform, and Econ lit - were searched. Titles/abstracts and full texts of articles were reviewed for eligibility; relevant articles were appraised for methodological quality. A best evidence synthesis was applied to determine key findings and generate recommendations based on an assessment of the quality, quantity, and consistency of evidence.

**Results:**

15,289 articles were identified from which 19 studies met eligibility criteria and were appraised as medium-to-high methodological quality. Fifteen studies focused on workers with a non-occupational injury or illness and only four focused on workers with an occupational injury or illness. There was strong evidence indicating that non-White and racial/ethnic minority workers were less likely to RTW following a non-occupational injury or illness when compared to White or racial/ethnic majority workers.

**Conclusions:**

Policy and programmatic attention should be directed towards addressing racism and discrimination faced by non-White and racial/ethnic minority workers in the RTW process. Our research also underscores the importance of enhancing the measurement and examination of race and ethnicity in the field of work disability management.

**Supplementary Information:**

The online version contains supplementary material available at 10.1007/s10926-023-10119-1.

## Purpose

Race and ethnicity may be differentiating markers of the return-to-work (RTW) experience for injured workers. Research shows inequities in occupational and non-occupational injury and illness across racial and ethnic groups; non-White (or, depending on the context, ‘ethnic minority’) workers face more frequent, severe, and disabling injuries and illnesses when compared to White workers [1–3]. RTW represents a series of clinical, policy and organizational activities that transition injured workers back to meaningful employment [4, 5]. Fostering optimal RTW practices represents a critical public health and social policy priority as it ensures that workers can re-enter the labor market following a health-related disruption and regain their financial livelihood, productivity, and well-being. Currently, it is unclear whether the RTW process differs according to race or ethnicity.

Among countries which collect and publish labor market data according to race and ethnicity, including the United States (US), United Kingdom (UK), and Canada, it is estimated that up to a quarter of the workforce consists of non-White workers [6–8]. Data also indicate that non-White workers experience significantly greater occupational morbidity [9]. For example, Seabury, Terp and Boden (2016) utilized two large US population-representative datasets to estimate the prevalence of work-related injury and disability by race and ethnicity [1]. Findings indicated that Black and Hispanic workers had a higher prevalence of injury and disability when compared to non-Hispanic White workers in models that adjusted for sex, age, and education. These findings have been replicated in other studies conducted in the US and in other developed labor markets [2, 3]. Research on a range of non-occupational chronic health conditions, such as cancer, cardiovascular disease and depression also indicates that non-White people are more likely to report severe disease symptoms, disability and mortality when compared to their White counterparts [1, 10–13].

Researchers have sought to examine the factors that contribute to racial and ethnic health inequities. Despite lacking a sound scientific evidence basis, the role of genetics has been routinely brought up as a primary factor that contributes to racial and ethnic differences in health outcomes inside and outside of the workplace. It is important to highlight that while genes might explain why an individual is susceptible to a disease, they cannot explain differences across social groups [13–16]. Indeed, race and ethnicity are social constructs associated with power and status that play a prominent role in shaping the distribution of social, economic, and health-related resources, including decent and safe work [17]. In cases of occupational morbidity and mortality, which are primarily attributed to workplace conditions, a genetic argument for racial differences cannot be entertained. Studies consistently find that non-White workers are disproportionately employed in occupations characterized by high physical and psychological job demands, and have hazardous working conditions and limited regulatory and labor protections when compared to White workers – a form of racial occupational segregation [12, 17–20]. Other research highlights that non-White people are exposed to a greater number of risk factors for disease and disability including lower levels of healthcare access, greater levels of stress, lower socioeconomic status, and more barriers to the performance of health-enhancing behaviours when compared to White people [11, 12, 21]. These inequities are widely understood to be the outcomes of systemic racism – or structures, policies, practices, and norms that disadvantage persons of color and contribute to racial health inequities [22, 23].

Despite the disproportionate burden of occupational and non-occupational injury and illness on non-White workers, it is unclear how race and ethnicity impact the RTW process. The RTW process is a series of steps that include injury reporting, seeking medical care and workers’ compensation benefits, accessing rehabilitation services, accommodation planning, work re-integration and stay-at-work [5, 24]. The RTW process occurs within a complex system that includes workplace, clinical and legal settings and a range of actors including workers and their employers, healthcare providers, workers’ compensation representatives and legislators [4, 25]. In an ideal scenario an injured worker will progress through RTW phases in a stepwise fashion [5]. However, some injured workers can face extended or intermittent work disability that results in significant employer and societal costs and can significantly disrupt a worker’s access to income and resources that are important to health and quality of life [26, 27]. Structural and interpersonal racism has the potential to operate throughout the RTW system and in a worker’s interactions with different actors and lead to adverse RTW outcomes [28]. Accordingly, racism may result in disparate RTW outcomes between workers of color and their White counterparts. Using a systematic review process, our study sought to assess whether racial and ethnic inequities contribute to differences in the RTW process among workers with an occupational or non-occupational injury or illness.

## Methods

We conducted a systematic review of the literature using a process developed by the Cochrane Collaboration which was adapted by the Institute for Work & Health (IWH) Systematic Review Program [29]. The review methods were registered with PROSPERO and met the 2020 Preferred Reporting Items for Systematic Reviews and Meta-analyses (PRISMA) statement guidelines.

### Literature Search

The search strategy followed a Population (P), Exposure (E), Comparator, (C) and Outcome (O) (PECO) framework and was designed to capture studies that examined the working population within OECD countries with an occupational or non-occupational injury or illness (P), non-White workers (E), a White worker comparison group (C) and measured any RTW outcome (O) (Table 1). To elaborate on the impact of discrimination according to ethnicity and race in different OECD contexts, we also included studies which compared racial/ethnic minority and majority groups. Research team members provided feedback and helped refine the search strategy. Database-specific controlled vocabulary terms and keywords were included and are available in Supplement 1. The terms within each category were combined using a Boolean OR operator and terms across the four main categories were combined using a Boolean AND operator. Medline (OVID), Embase (OVID), PsycINFO (OVID), CINAHL, Sociological Abstracts, ASSIA, ABI Inform, and Econ lit were searched for English, French or Spanish language articles that were published between January 2001-April 2021. Our decision to focus on articles published after 2000 was made to reflect the growing research on the impact racial and ethnic inequities can play in determining the health of the working population[30]. After removing duplicates, the search yields were combined and imported into the review software DistillerSR to facilitate relevance screening [31].

**Table 1:** Study inclusion and exclusion criteria using PECO framework

PECO category	Inclusion criteria	Exclusion criteria
**Population**	• Study included workers who experienced work disability due to an occupational or non-occupational injury or illness • Study sample based in an OECD labor market country context	• Study included immigrants or linguistic minorities where race or ethnicity was not specified • Study focused exclusively on military or veteran populations
**Exposure**	• Non-White worker • Racial or ethnic minority worker • Exposure to racism or discrimination related to race or ethnicity	• Studies where specific findings from non-White or racial/ethnic groups were not presented or where racism or discrimination related to race or ethnicity was not collected
**Comparison**	• White workers • Racial or ethnic majority workers	• No comparison presented
**Outcome**	• Any outcome measured reflecting the RTW process	• RTW outcomes were not presented • Studies where pre-injury or illness employment were not presented

### Relevance Screen

Relevancy screening was informed by our PECO framework. Also, articles were included if they involved primary research, were published ≥ 2001, focused on workers in OECD countries who experienced work disability from an occupational or non-occupational injury or illness, collected information on the race or ethnicity of study participants and measured any dimension of RTW. We included quantitative study designs where RTW outcomes were examined according to race or ethnicity and where a statistical effect estimate was reported. Studies were excluded if they were secondary research, commentaries, editorials, or case studies.

Relevance screening occurred over two steps. First, titles and abstracts of references identified in the search were divided among research team members and were screened independently by two reviewers for relevancy. Due to the large number of studies retrieved by the search, the artificial intelligence (AI) capabilities of DistillerSR were used for title and abstract screening. To train the AI, the first 15% of titles and abstracts were screened independently by two human reviewers to identify studies of relevance to the review. Once the AI was trained, it served as a secondary reviewer to a single human reviewer. Any disagreements between a human reviewer and the AI were resolved by a third (human) reviewer. Articles that met relevance at the first level of screening were carried forward for a full-text review, which was carried out by two human reviewers. Disagreements between the two reviewers were discussed in team meetings. Relevance decisions on title and abstract and full-text screening demonstrated high moderate to high inter-rater reliability, suggesting that reviewers were consistently applying inclusion/exclusion criteria to the screening processes. Reference lists of articles that were examined during full-text review were checked to ensure no relevant articles were missed.

### Quality Appraisal and data Extraction

A modified version of a quality assessment tool developed by IWH for systematic reviews in the field of work disability management was used to conduct quality appraisal (Supplement 2). The quality assessment tool consisted of 15 questions that examined internal, external, and statistical validity of each article through an assessment of study design and objectives, recruitment procedures, outcome and exposure measurement and analysis. Questions were added to the tool by the research team to examine the quality of measurement of race and ethnicity and assess sample heterogeneity. Using the tool, each relevant article was appraised by two independent reviewers. A final weighted sum score of the quality criteria was generated and converted to a percentage score. Using the percentage score, studies were categorized as high (≥ 85%), medium (50–84%) or low quality (< 50%). Research team members held meetings to reach consensus on final appraisal scores and rankings. Only studies appraised as high and medium quality were utilized in the evidence synthesize phase. Data were extracted from relevant articles to create summary tables that presented sample description and racial and ethnic breakdown of the study sample, study design, and RTW outcomes.

### Evidence Synthesis

Given that studies varied in their length of observation, design, sample characteristics, confounding variables collected and RTW outcomes measured, pooled effect estimates were not calculated. Instead, we undertook a best evidence synthesis approach to examine the impact of race and ethnicity on RTW and generate applied practice messages based on available evidence [32]. Evidence was synthesized to determine levels of evidence using an algorithm that considers the methodological quality (i.e., high, medium, or low methodological quality) and quantity of studies (i.e., number of studies with similar sample characteristics and outcome measures) and the consistency of study findings (i.e., number of studies which produce similar findings) (Table 2). As an example, findings with a strong level of evidence are those where there is a minimum of three high quality studies which have consistent findings. Refer to Table 2 for additional details on how the best evidence synthesis was utilized to determine levels of evidence. According to the best evidence synthesis approach,  with a strong level of evidence specific recommendations for policy and practice can be generated. Results supported by a moderate level of evidence contribute to practice considerations. On the other hand, limited, mixed or insufficient evidence levels contribute to a lack of evidence to guide policies or practices.

**Table 2:** Best evidence synthesis algorithm for messages

Level of evidence	Minimum quality	Minimum quantity	Consistency	Strength of message
Strong	High^a^ (H)	3	3 H agree; if 3^+^ studies, ≥3/4 of the M and H agree	Recommendations
Moderate	Medium^b^ (M)	2 H *or* 2 M and 1 H	2 H agree *or* 2 M and 1 H agree; if 3^+^studies, ≥2/3 of the M and H agree	Consideration
Limited		1 H or 2 M *or* 1 M and 1 H	2 (M and/or H) agree; if 2 studies, > 1/2 of the M and H agree	Not enough evidence to make recommendations or considerations
Mixed		2	Findings are contradictory	
Insufficient	Medium quality studies that do not meet above criteria			

*Notes*: ^a^High (H) quality study =  ≥85% in quality appraisal; ^b^Medium (M) quality study = 50–84% in quality appraisal

## Results

**Fig. 1:**
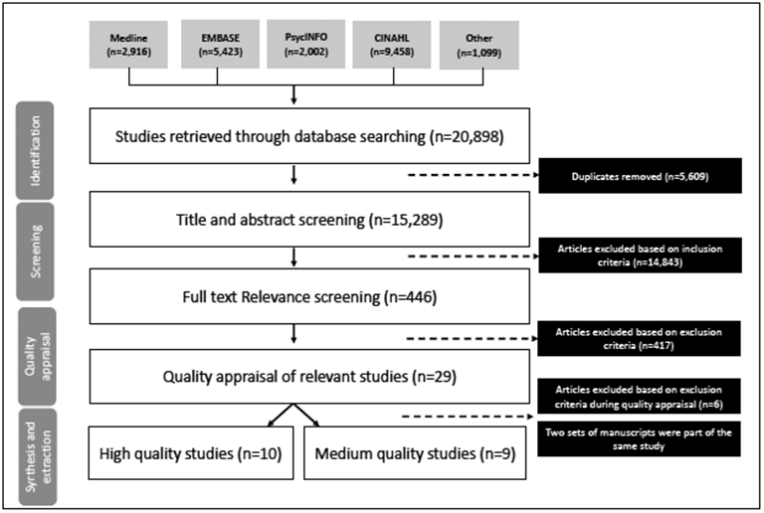
Flow chart of study identification, selection, and synthesis

Our search yielded 15,289 articles that were published between 2001 and 2021 after duplicates were removed. After title/abstract screening, 446 articles met initial selection criteria and were carried forward for full-text review. A total of 23 studies were found to meet eligibility criteria. Four sets of studies were grouped together as they utilized the same study sample and asked similar study questions [32–39]. Using the quality appraisal tool, ten studies were of high quality (≥ 85% quality appraisal score) and nine articles were of medium quality (50–84% quality appraisal score). No articles were appraised as being of low quality (< 50% quality appraisal score) (see Fig. 1). When compared to high quality studies, those that were appraised as medium quality had poorly defined recruitment procedures, low study participation rates, were limited in their assessment of race or ethnicity, did not report sample characteristic differences between participants and non-participants and did not collect information on important confounders.

### Study Descriptions

A description of studies is presented in Table 3. Most studies were conducted in the US (n = 16); the remaining were conducted in the UK (n = 1) and Israel (n = 2). Fifteen studies focused on workers with non-occupational injury or illness including spine injury (n = 3), cancer (n = 3), limb trauma (n = 2), burn injury (n = 2) and stroke (n = 2). Of the four studies on workers with occupational injury or illness, most focused on low back pain (n = 3) and one examined a broad cohort of compensation claimants. In most studies, White workers were the comparison group (n = 15). An examination of the racial and ethnic breakdown of participants in the studies revealed limited heterogeneity. Thirteen studies included Black, Black non-Hispanic or African American samples (hereafter, collectively referred to as Black) (n = 13). Several studies included samples who were categorized as non-White or other race (n = 8). Eight studies included Hispanic, Latinx or Mexican Americans participants (hereafter, collectively referred to as Latinx). One study consisted of participants who were categorized as American Indian and Alaskan Native, Asian, multiracial and Native Hawaiian and Pacific Islander. Two sets of studies conducted in Israel examined RTW differences between Jewish Israeli and racial/ethnic minority workers including Arab Israelis, immigrants from Soviet Union, and Ethiopian immigrants.

**Table 3:** Description of studies identified in our systematic review of evidence

Author, Year	Region	Study objectives	Study design	Sample size and brief description	Length of observation	Racial/ethnic groups (n [%])^*^	Injury or illness type	Outcome	Quality
Asher AL, et al., 2017	United States of America (US)	To create a predictive model of patients’ ability to return to work (RTW) following lumbar spine surgery	Prospective cohort study	• 4,694 • Patients undergoing elective spine surgery for degenerative lumbar disease	Three months post initial surgery	• White (4,246 [91%]) • African American (302 [6.0%]) • Other race (146 [3.0%])	Non-occupational	• RTW	Medium
Ben-Shalom Y, Mamun AA., 2015	US	To study factors associated with achieving RTW milestones	Retrospective cohort study	• 417,238 • A representative proportion of disability insurance beneficiaries from 1996 to 2004	Five years after receipt of first disability insurance benefit	• White non-Hispanic (294,153 [71%]) • Black non-Hispanic (76,355[18%]) • Hispanic (28,372 [6.8%]) • Other or unknown race (17,941 [4.3%])	Non-occupational	• Enrollment in employment services • Start of trial work period • Trial work period completion • Suspension or termination of benefits due to RTW	High
Blinder V, et al., 2013 (a) Blinder V, et al., 2012 (b)	US	To identify early correlates of not returning to work (a) or not being employed (b) for low-income women treated for breast cancer	Prospective cohort study	• 274 (a) / 290 (b) • Women employed at time of a breast cancer diagnosis and were uninsured or underinsured and have a family income ≤200% of federal poverty level	Six, 18-, and 36- and 60-months following breast cancer diagnosis	• Latina (a:145 [53%]) (b:179 [62%]) • Non-Latina white (a:90 [33%] (b:111 [38%])	Non-occupational	• Not returning to work (a)/ not reporting employment (b)	Medium
Bradley CJ, Wilk A., 2014	US	To examine differences between African American and non-Hispanic white women in employment and change in work hours in women newly diagnosed with breast cancer who were initially employed and insured	Prospective cohort study	• 548 • Employed women diagnosed with breast cancer within two months of initiating treatment with intent to cure	Two- and nine-months following baseline interview	• Non-Hispanic white (429 [78%]) • African American (119 [22%])	Non-occupational	• Employment status • Hours worked/week	Medium
Busch MA, et al., 2009	United Kingdom (UK)	To investigate the frequency and determinants of return to paid work after stroke in a multi-ethnic urban population	Prospective cohort study	• 400 • Patients in a stroke registry with first ever stroke between January 1995 and December 2004 and working immediately before stroke	12 months following stroke	• White (231 [59%]) • Black (128 [33%]) • Other (31 [8.0%])	Non-occupational	• Employment status	Medium
Carrougher GJ, et al., 2020	US	To investigate the effect of patient and injury characteristics on employment for working-aged adult survivors of burn injury	Prospective cohort study	• 967 • Survivors of burn injury, ≥18 years of age or 18 years at follow-up, inpatient hospitalization of ≥ 3 days, known pre-burn employment status	12 months following burn injury	**Race** • White (654 [78%]) • Black (127 [15%]) • American Indian/Alaska Native (24 [2.9%]) • Asian (15 [1.8%]) • Other (9 [1.0%]) • Multiracial (6 [0.70%]) • Native Hawaiian/Pacific Islander (3 [0.40%]) **Ethnicity** • Hispanic/Latino (157 [17%]) • Non-Hispanic/non-Latino (761 [83%])	Non-occupational	• Employment status	Medium
Chibnall JT, Tait RC., 2009	US	To investigate sociodemographic, claim process and short-term adjustment predictors of long-term clinical adjustment among workers’ compensation claimants with low back pain	Prospective cohort study	• 374 • Workers’ compensation claimants with low back pain whose claims were settled in Missouri between January 1, 2001 and June 2, 2002	Six years post workers’ compensation settlement	• Caucasian (203 [61%]) • African American (171 [52%])	Occupational	• Employment status • Receipt of social security disability insurance	High
Chibnall JT, et al., 2005 (a) Tait RC, et al., 2004 (b)	US	To examine relationships among race, socioeconomic status, and post-settlement outcomes (a) and case management (b) of occupational back injuries	Cross-sectional study	• 1,472 • First-incident workers’ compensation claimants with low back injuries whose claims were settled between January 1, 2001 and June 1, 2002	Not reported	• Caucasian (892 [61%]) • African American (580 [39%])	Occupational	• Financial struggle (a) • Claim duration (injury to settlement) (b) • Receipt of temporary disability payment (b)	High
Friedman LS, Ruestow P, Frost L., 2012	US	To assess ethnic disparities in monetary compensation among construction workers injured on the job	Retrospective cohort study	• 1,039 • Construction workers who filed a workers’ compensation claim in Illinois between 2000 and 2005	Not reported	• White (724 [70%]) • Hispanic (168 [16%]) • Other (79 [7.6%] • Black (68 [6.5%])	Occupational	• Total workers’ compensation claim ($USD) • Mean temporary work disability (weeks)	High
Khan I, et al., 2019	US	To investigate the factors associated with RTW in patients who achieved otherwise favorable outcomes after lumbar spine surgery	Retrospective cohort study	• 12,435 • Patients undergoing lumbar surgery and were employed prior to surgery and completed 12-month follow-up	12 months post-surgery	• Caucasian (11,016 [90%]) • African American (886 [7.2%]) • Other race (353 [2.9%])	Non-occupational	• RTW	High
MacKenzie EJ, et al., 2006	US	To understand the factors that influence RTW after major limb trauma	Prospective cohort study	• 432 • Patients admitted to one of eight level 1 trauma centres for treatment of a severe lower extremity injury • English or Spanish speaking and aged 18–69 years	Three, six, 12-, 24- and 84-months post-injury	• White† • Non-white†	Non-occupational	• RTW	Medium
Marom BS, et al., 2020 (a) Marom BS, et al., 2018 (b)	Israel	To determine the time to RTW and to examine the effect of ethnicity and other prognostic variables on RTW among male manual workers who had experienced a hand injury	Prospective cohort study	• 178 • Male participants, aged 22–65 years who sustained acute hand injury below the elbow, either during or outside of working hours • Manual workers prior to hand injury and were referred by a physician to one of seven occupational therapy clinics • Read or write in Arabic or Hebrew language	12 months post-injury	• Jewish (88 [50%]) • Arab (90 [51%])	Non-occupational	• Time to RTW (a) • Employment status (b)	Medium
Meade MA, et al., 2004	US	To examine issues of employment and race for persons with spinal cord injury	Retrospective cohort study	• 5,925 • Participants who experienced a spinal cord injury between 1972–2002 and part of a larger database • Classified as African American or white • Ages 18–65 years at time of injury	One-year post-injury (multivariable model) Five, ten, 15-, and 20-years post-injury (descriptive analysis)	• White (4,210 [71%]) • African American (1,715 [29%])	Non-occupational	• Employment status	High
Pham TN, et al., 2020	US	To examine the association of extremity contractures with employment after burn injury	Retrospective cohort study	• 1,203 • Participants aged 18–64 years identified in a burn database (1994–2003) who were working prior to injury	Six-, 12- and 24-month post-injury	• Caucasian† • Non-Caucasian†	Non-occupational	• RTW	Medium
Sanchez KM, Richardson JL, Mason HRC, 2005	US	To assess the role of person, disease, and work-related factors on a timely return to the workplace among employed colorectal cancer survivors who sustained employment five years after initial cancer diagnosis	Cross-sectional study	• 142 • Participants who survived a colorectal cancer diagnosis between 1994 and 1995 were randomly selected from two population-based cancer registries • Speak and read English or Spanish • Ages 30–59 years at the survey		• White (104 [73%]) • Black (22 [16%]) • Hispanic (16 [14%])	Non-occupational	• Delayed RTW (> 2 months following diagnosis)	High
Savitsky B, et al., 2020 (a) Savitsky B, et al., 2020 (b)	Israel	To examine socioeconomic and other predictors of RTW (a) and duration to RTW (b)	Retrospective cohort study	• 44,740 (a) / 45,291(a) • Resident to Israel and aged 21–67 years • Injured and hospitalized between January 1, 2008 and December 31st 2013 • Employed 2 months prior to injury as a salaried worker•	One month, one year and 2 years post-injury	• Other Israelis (26,858 [59.3%]) • Israeli Arabs (11,232 [24.8%]) • Immigrants from former Soviet Union (6,341 [14%]) • Ethiopian Immigrants (679 [1.5%]) • Missing (181[0.4%])	Occupational and non-occupational	• Non-RTW	High
Skolarus LE, et al., 2016	US	To compare the proportion of stroke survivors who returned to work within 90 days following their stroke by ethnicity and investigate the role of sociodemographic factors and stroke severity	Prospective cohort study	• 125 • Stroke patients from a larger population-based surveillance study stroke survivors (August 2011-December 2013) who were employed at the time of their stroke	90 days post-stroke	• Mexican Americans (71 [57%]) • Non-Hispanic white (54 [43%])	Non-occupational	• RTW	Medium
Strong LL, Zimmerman FJ, 2005	US	To examine the relationship between race/ethnicity and the number of workdays missed owing to an injury or illness that caused a participant to miss work according to gender	Prospective cohort study	• 35,710 • Cohort drawn from ten waves of data (years 1988–2000) from a nationally representative population-level dataset	Not reported	• Non-Hispanic white (20,377 [57%]) • Hispanic (5,387 [15%]) • African American (9,946 [28%])	Non-occupational	• Workdays missed due to injury or illness	High
Tait RC, Chibnall JT, 2000	US	To examine medical and psychosocial factors associated with work injury management decisions for patients with low back pain	Retrospective cohort study	• 132 • Workers’ compensation claimants with refractory low back injuries whose impairment was physician assigned	Not reported	• Caucasian (84 [64%]) • African American (47 [36%])	Occupational	• Temporary total disability costs related to excused time off work	High

**Notes**: * = Racial groups presented based on the study author’s classification; † = author did not report a breakdown of racial groups included in their study

As a primary outcome measure, most studies measured whether workers reported RTW or were employed following injury or illness (n = 13). Other outcomes uncovered in our review included work disability duration (n = 4), receipt of income support following injury or illness (n = 3), total cost and length of workers’ compensation claim (n = 3), hours worked/week following injury or illness (n = 1), involvement in RTW services (n = 1), and financial struggle following injury or illness (n = 1).

### Race, Ethnicity and RTW Outcomes

As presented in Table 4, studies consistently showed that non-White or racial/ethnic minority groups reported adverse RTW outcomes following an occupational or non-occupational injury or illness when compared to White or racial/ethnic majority workers.

**Table 4:** Key study findings related to race and ethnicity and results of quality appraisal assessment

Author∼, Year	Racial/ethnic groups*	Findings ^‡^	Quality
Asher AL, et al., 2017	• White • African American • Other race	• African American participants significantly less likely to RTW following lumbar spine surgery compared to white participants (Hazard ratio [HR] = 0.71; 95% confidence interval [95% CI] 0.62–0.82) • Other race groups were significantly less likely to RTW following lumbar spine surgery compared to white participants (HR = 0.82; 95% CI 0.67-1.0)	Medium
Ben-Shalom Y, Mamun AA, 2015;	• White non-Hispanic • Black non-Hispanic • Hispanic • Other or unknown race	• Black disability insurance beneficiaries more likely to be enrolled in employment services compared to those who were non-Hispanic white (β = 0.014, p < 0.001) • Black disability insurance beneficiaries more likely to start (β = 0.026, p < 0.001) and complete (β = 0.023, p < 0.001) trial work period compared to those who were white • Hispanic disability insurance beneficiaries less likely to be enrolled in employment services when compared to those who were non-Hispanic white (β= -0.0080, p < 0.05) • Other or unknown race disability insurance beneficiaries less likely to be enrolled in employment services when compared to non-Hispanic white participants (β= -0.011, p < 0.001)	High
Blinder V, et al., 2013 (a) Blinder V, et al., 2012 (b)	• Latina • Non-Latina white	• Latina participants more likely to report not returning to work (a)/not being employed at only six months (Odds ratio [OR] = 0.31, 95% CI 0.12–0.81) and 18 months (OR = 0.73, 95% CI 0.27-2.0) following breast cancer diagnosis when compared to non-Latina white participants (b) • The difference between Latina participants and non-Latina participants was not significantly different at 36 months (53% vs. 59%) (b)	Medium
Bradley CJ, Wilk A., 2014	• Non-Hispanic white • African American	• African American participants with breast cancer significantly less likely to be employed at two months following cancer diagnosis (69%) when compared to non-Hispanic white participants (85%) (Odds ratio [OR] = 0.43, 95% CI 0.26–0.71) • African American participants with breast cancer more likely to report fewer work hours at nine months following baseline survey (-4.3%) when compared to non-Hispanic white participants (-2.9%) (β= = -2.1, p < 0.05)	Medium
Busch et al., 2009	• White • Black • Other	• Black participants less likely to be employed 12 months following a stroke (30%) when compared to white participants (OR = 0.41; 95% CI 0.19–0.88)	Medium
Carrougher GJ, et al., 2020	**Race** • White • Black • American Indian/Alaska Native • Asian • Other • multiracial • Native Hawaiian/Pacific Islander **Ethnicity** • Hispanic/ Latino • Non-Hispanic/ non-Latino	• White race, non-Hispanic ethnicity participants with a burn injury were more likely to be employed at 12 months than those who were non-white race or Hispanic ethnicity (OR = 1.6, 95% CI 1.1–2.3)	Medium
Chibnall JT, Tait RC, 2009	• Caucasian • African American	• Caucasian participants with low back pain (67%) were significantly more likely than African American participants to be employed six years post workers’ compensation settlement (57%) when compared to (OR = 1.6 95% CI 1.0- 2.5) • African American participants with low back pain were more likely to receive Social Security Disability Insurance (39%) when compared to Caucasian participants (25%) (OR = 2.0 95% CI 1.3–3.2)	High
Chibnall JT, et al., 2005 (a) Tait RC, et al., 2004 (b)	• Caucasian • African American	• African American participants with occupational back injuries were significantly more likely to report post-settlement financial struggle compared to Caucasian participants (β = 0.12, p < 0.001) (a) • African American participants with occupational back injuries reported shorter claim periods compared to Caucasian participants (18.2 months vs. 23.2 months) (β = -0.14, p < 0.0001) (b) • African American participants with occupational back injuries were less likely to receive a temporary disability payment (21%) compared to Caucasian participants (42%) (OR = 0.45, 95% CI 0.30–0.60) (b)	High
Friedman LS, Ruestow P, Frost L., 2012	• White • Hispanic • Other race • Black	• Black ($47,935.00), Hispanic ($48,519.00) and other race ($43,048.00) participants awarded lower total mean monetary workers’ compensation settlements following an injury compared to white participants ($60,431.00) • Black (21.8 days) Hispanic (26.7 days) participants reported shorter mean temporary work disability days than white participants (29.5 days)	High
Khan I, et al., 2019	• Caucasian • African American • Other race	• African American participants (6.2%) were significantly less likely to RTW following lumbar spine surgery when compared to Caucasian participants (91%) (OR = 0.60, 95% CI 0.50–0.80) • Among those with favorable surgical outcomes, there was no significant RTW difference between Caucasian, African American participants, and other racial participants (X^2^ = 1.1, P = 0.059)	High
MacKenzie EJ, et al., 2006	• White • Non-white	• White participants were significantly more likely to RTW at 84 months post major limb trauma when compared to non-white participants (Relative Rate Ratio [RR] = 1.8, 95% CI 1.2–2.7)	Medium
Marom BS, et al., 2020 (a) Marom BS, et al., 2018 (b)	• Jewish • Arab	• No significant difference between Arab (71%) and Jewish (80%) participants when comparing time to RTW following hand injury (HR = 1.2, 95% CI 0.76-2.0) (b) • Difference between Arab (29%) and Jewish participants (46%) in employment status at three months following hand injury was not significant when adjusting for legal counsel, educational attainment, and disability severity (OR = 1.1, 95% CI 0.46–2.5) (a)	Medium
Meade MA, et al., 2004	• White • African America	• African American participants were significantly less likely to be employed at 12 months (5.9%) post-spinal cord injury when compared to white participants (16%) • The racial difference in employment following spinal cord injury persisted across the descriptive follow-up period (β=-1.2, p < 0.001)	High
Pham TN, et al., 2020	• Caucasian • Non-Caucasian	• Non-Caucasian ethnicity was significantly associated with a lower likelihood of RTW when compared to Caucasian participants after burn injury (period post-follow-up not reported) (OR = 0.16–0.35 (depending on ethnicity), 95% CI 0.040–0.89)	Medium
Sanchez KM, Richardson JL, Mason HRC, 2005	• White • Black • Hispanic	• Black (OR = 1.08, 95% CI 0.41–2.81) and Hispanic (OR = 1.47, 95% CI 0.50–4.27) ethnicity not significantly associated with delayed RTW following colorectal cancer diagnosis when compared to white participants	High
Savitsky B, et al., 2020 (a) Savitsky B, et al., 2020 (b)	• Other Israelis • Israeli Arabs • Immigrants from former Soviet Union • Ethiopian Immigrants	• Israeli Arabs significantly more likely to report non-RTW at one-month post-injury (53%) when compared to the other Israeli group (33%) (OR = 1.6, 95% CI 1.5–1.7) • There was no significant difference in the likelihood non-RTW at one-month post-injury when comparing Ethiopian Immigrants (43%) and the other Israeli group (33%) (OR = 0.96, 95% CI 0.81–1.14) • Israeli Arabs significantly more likely to report non-RTW at one-year post-injury (20%) when compared to the other Israeli group (8.8%) (OR = 1.9, 95% CI 1.8–2.1) • There was no significant difference in the likelihood non-RTW at one-year post-injury when comparing Ethiopian Immigrants (14%) and the other Israeli group (8.8%) (OR = 1.22, 95% CI 0.97–1.14) • Israeli Arabs significantly more likely to report non-RTW within 2 years post-injury (14%) when compared to the other Israeli group (5.7%) (OR = 2.1, 95% CI 1.9–2.3) • There was no significant difference in the likelihood non-RTW at one-year post-injury when comparing Ethiopian Immigrants (9.6%) and the other Israeli group (5.7%) (OR = 1.25, 95% CI 0.92–1.70) • Israeli Arabs (HR = 1.4, 95%CI 1.3–1.4) and Ethiopian immigrants (HR = 1.2, 95% CI 1.1–1.3) experience a longer duration of stay out of work compared to other groups of Israeli workers	High
Skolarus LE, et al., 2016	• Mexican Americans • Non-Hispanic white	• Mexican American participants were significantly less likely to RTW following a stroke (31%) when compared to non-Hispanic white participants (50%) (OR = 0.85, 95% CI 0.32–2.2) • Relationship between being Mexican American being less likely to RTW was non-significant in model which also included educational attainment and level of impairment	Medium
Strong LL, Zimmerman FJ, 2005	• Non-Hispanic white • Hispanic • African American	• Hispanic male workers missed more mean workdays (38 days) due to injury or illness than male non-Hispanic white workers (28 days) but the relationship was not significant at the multivariable level (OR = 1.1, 95% CI 0.84–1.5); No significant differences were observed between Hispanic and non-Hispanic white female workers • African American male (30.9 days) missed more mean workdays due to injury or illness than non-Hispanic white male (28.1 days) (OR = 0.83, 95% CI 0.64–1.1) • African American female workers missed more mean workdays (45.2 days) due to injury or illness than non-Hispanic white female workers (24.0 days) but the relationship was not significant at the multivariable level (OR = 1.0, 95% CI = 0.77–1.4)	High
Tait RC, Chibnall JT, 2000	• Caucasian • African American	• In the absence of legal representation, African American participants indicated significantly lower temporary total disability costs related to low back pain when compared to Caucasian participants (F (1, 126) = 4.4, p < 0.05). This relationship did not hold in the presence of legal representation.	High

**Notes**: * = As described by authorship team; ~ = presented in alphabetical order; ‡ = Findings presented are those from finalized multivariable models presented in the author’s manuscript; CI = confidence interval

#### RTW or Employment Status Following Injury or Illness

Non-White and racial/ethnic minority workers were less likely to report returning to work or being employed following an occupational or non-occupational injury or illness when compared to White and racial/ethnic majority workers. Seven studies indicated that Black workers were less likely to report RTW or being employed following an injury or illness when compared to White workers [35, 39–45]. One study found that the relationship between race and RTW was statistically significant among those reporting poor quality of surgical outcomes. In this study, no differences in RTW existed between Black or other race participants and White participants among those with favorable surgical outcomes [44]. Also, when compared to White workers, studies found that non-White (n = 2), other race (n = 1), and Latinx (n = 4) workers were less likely to RTW[33, 34, 40, 43, 45–48]. Studies from Israel highlighted mixed findings. One study found that Arab Israelis were less likely to report RTW at one-month, one-year and 2-years post injury when compared to Jewish Israelis [38, 39]. Another study found that, while differences between Arab and Jewish Israeli workers existed in the likelihood of returning to work, the relationship was not significant when controlling for educational attainment, disability severity or access to legal counsel [36, 37].

#### Cost and Length of Workers’ Compensation Claim

Two studies indicated that Black workers experiencing an occupational injury or illness were more likely to report a shorter claim duration when compared to their White counterparts [48–51]. Similarly, Latinx, and other race workers experiencing an occupational injury reported shorter claim durations when compared to White workers with a workplace injury [50, 51]. Additionally, total awarded workers’ compensation settlement following an occupational injury were less for Black, Latinx, and other race workers when compared to White workers [51]. One study of workers’ compensation claimants in the US found that in the absence of legal representation, Black workers indicated lower temporary total disability costs related to low back pain when compared to White workers [52].

#### Length of Disability Absence

One study using a large US-based population-level dataset found that Black male workers were more likely to report more missed workdays due to injury or illness when compared White workers. On the other hand, no significant difference existed between Black women when compared to White women. The same study found that Latinx men experienced more missed workdays when compared to White men, but this difference was not statistically significant in multivariable models [53]. Another study found that Arab Israelis and Ethiopian Immigrants in Israel experienced a longer disability duration when compared to Jewish Israeli workers [38, 39].

#### Income Support

Studies of workers experiencing a work disability due to an occupational injury or illness found that Black workers were more likely to receive social security disability insurance, or a temporary disability payment compared to White workers [35, 49, 50].

#### Work Hours Following RTW

One study indicated that Black participants worked fewer hours following a non-occupational injury when compared to White participants [41].

#### Financial Struggle

A single study found that Black workers injured on the job were more likely to report post-settlement financial struggle when compared to White workers [49, 50].

#### Involvement in RTW Services

Only one study examined the relationship between race and involvement in RTW services [54]. In a study of disability insurance beneficiaries, Black and Latinx participants were more likely to be enrolled in employment services when compared to White participants. Additionally, Black participants were more likely to start and complete a trial work period when compared to White participants [54].

### Evidence Synthesis

For each RTW outcome identified in our review results were synthesized to generate key messages and practice recommendations when an adequate quantity and quality of studies existed (Table 5). Findings were first pooled broadly to compare differences between non-White or racial/ethnic minority workers and White or racial/ethnic majority workers. A strong level of evidence existed which highlighted that non-White or racial/ethnic minority workers were less likely to RTW following a non-occupational injury or illness when compared to White or racial/ethnic majority workers (3 high quality studies [H] and 7 medium quality studies [M]). Of note, from the samples of studies there were a sufficient quantity of studies focusing specifically on Black and Latinx workers to synthesize evidence. Findings from these studies indicated that Black worker (moderate levels of evidence; 2 H and 3 M) and Latinx workers (limited levels of evidence; 1 H and 1 M) were less likely to report RTW when compared to White workers following a non-occupational injury or illness. Limited, mixed, or insufficient evidence exists on racial or ethnic inequities on the other RTW outcomes identified and corresponding practice messages could not be developed. Of note, there was insufficient evidence examining racial or ethnic differences across RTW outcomes for studies focusing on workers with occupational injuries or illnesses.

**Table 5:** Study messages based on levels of evidence

Outcome	Levels of evidence	Number of High [H] and Medium [M] quality studies	Message
**Key messages for non-White or racial/ethnic minority groups** ^‡^			
Returning to paid employment following a non-occupational injury or illness*	Strong	3 H, 7 M	Non-White or ethnic minority workers are less likely to report RTW following a non-occupational injury or illness and will benefit from specific policy and programmatic attention
Enrollment in RTW programming or employment services	Limited	1 H	
Receipt of social security or disability benefits following occupational injury or illness	Mixed	2 H	Not enough evidence from the scientific literature due to mixed findings comparing non-White or ethnic minority workers and receipt of social security or disability benefits
Claim duration following occupational injury or illness	Insufficient	1 H, 1 M	Not enough evidence from the scientific literature
Total workers’ compensation claim ($) following occupational injury or illness		1 H	
Financial struggle following occupational illness		1 H	
Total missed workdays following non-occupational injury or illness		1 H	
RTW following occupational injury or illness		1 M	
Starting and completing trial work periods following non-occupational injury or illness		1 M	
Total work hours following non-occupational injury or illness		1 M	
**Key messages for specific racial and ethnic minority groups** ^**†**^			
Returning to paid employment following a non-occupational injury or illness^*^	Moderate	Black workers; 2 H, 3 M	Black workers may face obstacles to returning to work following non-occupational injury or illness and could benefit from policy and programmatic attention
Returning to paid employment following a non-occupational injury or illness^*^	Limited	Latinx workers; 1 H, 1 M	

**Notes**: ‡ = non-White included any persons of color or ethnic minority workers; * = Returning to paid employment following a non-occupational injury or illness includes a combination of return-to-work and employment status outcomes; RTW = return-to-work; H = high quality study, M = medium quality study; † = specific racial and ethnic subgroup analysis for findings with strong levels of evidence

## Discussion

Examining and addressing the barriers to RTW following injury or illness represents a critical labor market priority and has important population health implications. Our study sought to examine the relationship between race and ethnicity and RTW. Despite the disproportionate burden of occupational and non-occupational injury or illness faced by non-White and minority ethnic workers highlighted in past research [1–3], our systematic review showed that race and ethnicity have been underexplored when it comes to RTW. The research we uncovered highlighted disadvantages faced by non-White and racial/ethnic minority workers in returning to work after a non-occupational injury or illness. Strategies addressing the unique RTW obstacles faced by non-White and ethnic minority workers re-entering employment following an injury or illness are critical. Our research also highlights the importance of enhancing the measurement and examination of race and ethnicity in the work disability management field.

Research has consistently highlighted that non-White workers report more frequent and severe occupational injury or illness when compared to their White counterparts [1–3]. Racism and discrimination have been at the heart of these health inequities and represent structural determinants that shape physical and mental health among non-White and racial/ethnic minority workers [22, 23]. To our knowledge, this systematic review is the first of its kind to synthesize evidence concerning the impact of racial and ethnic inequities on the RTW process. Our research uncovered 19 published studies that examined racial and ethnic difference in RTW. The relatively small number of studies offers a limited evidence base that can inform the design of organizational work disability management programs and policies to meet the needs of non-White and minority racial/ethnic workers. It is also important to highlight that most studies in our review examined RTW among workers with a non-occupational injury or illness. Less information was available on the experiences of those injured at work. Also, limited research addressed a broader range of RTW outcomes including healthcare utilization, access to workers’ compensation and income support, accommodation planning and stay-at-work. It is recommended that data on race and ethnicity be routinely collected to enhance our understanding of the challenges faced by workers across different phases of the RTW process and to enhance strategies that address inequities in work disability management that are related to race and ethnicity [55].

A key finding from our study was that non-White and racial/ethnic minority workers with a non-occupational injury were less likely to RTW when compared to White or majority racial/ethnic workers. When looking at specific racial groups, our review found a moderate level of evidence suggesting that Black workers with a non-occupational injury or illness were less likely to RTW when compared to White workers. Our findings align with a body of evidence which show disadvantage faced by Black and non-White people in accessing care and social policy supports stemming from racism and discrimination [55–58]. Unclear from the available research is an explanation of how racial inequities specifically impact RTW. Non-White workers may be more likely to be exposed to a system of forces that contribute to more severe and disabling injury or illness and create obstacles to accessing healthcare access and other resources that support work re-entry when compared to their White counterparts [23]. Findings can also be contextualized through the theory of racial capitalism which describe racism as an underlying dimension of the design of capitalist labor markets [59]. Racial divisions of labor can reinforce adverse working conditions for non-White and racial/ethnic minority workers and mean that they are more likely to work in demanding and less supportive employment environments when compared to White or racial/ethnic majority workers. Through the lens of racial capitalism, it can be posited that non-White workers are more likely to be exposed to work disability managements systems that are disadvantageous and may make returning to work more challenging [60, 61]. More research is required to understand the mechanisms by which racial inequities may contribute to barriers at different phases of the RTW process.

Through the application of a best evidence synthesis approach, our systematic review points to the importance of designing policies and programs which address the RTW challenges of non-White and racial/ethnic minorities with a non-occupational injury or illness. To address racial and ethnic inequities in RTW, an anti-racist approach to work disability management may be adopted [62]. Anti-racism interventions are defined as action-oriented educational or policy-level strategies that identify racial inequities and make systemic and political change to addresses interlocking systems of social oppression [23]. Increasingly, anti-racism interventions are being applied to diverse healthcare settings to address the multiple pathways in which racism can impact access to healthcare services, including individual (e.g., training on providing culturally competent care), community (e.g., actively engage non-White and racial/ethnic minority communities in program planning and delivery), organizational (e.g., leadership towards anti-racist strategic goals) and policy change (e.g., meaningful involvement of non-White and racial/ethnic minority people in policy change) [63]. An anti-racist approach can provide a multifaced and action-oriented view for work disability management professionals to address the diverse structural challenges faced by non-White and minority racial/ethnic workers in the RTW process.

Strengths of this review included the utilization of a rigorous systematic review methodology that was designed specifically for the field of work disability management. There were also limitations that should be considered. To address our research objectives, we included peer-reviewed quantitative studies and excluded qualitative studies and gray literature. Additional research is required to synthesize other forms of evidence to enhance our understanding of the RTW experience following an occupational or non-occupational injury or illness for non-White and ethnic minority workers and to expand on the role of racism and discrimination. We utilized a quality appraisal tool to evaluate the internal, external, and statistical validity of each study. At the same time, the tool may have been limited in its ability to assess the measurement of race and ethnicity. It is recommended that quality appraisal tools be enhanced through the design and inclusion of questions which appropriately assess the racial and ethnic diversity of study samples and quality of measurement of racism and discrimination.

RTW following an injury or illness is a complex process that can be shaped by worker characteristics and contextual factors. Our systematic review brings to the forefront the role that race and ethnicity play in the RTW process and the structural conditions that can disadvantage different worker groups. RTW inequities for non-White and minority racial/ethnic workers experiencing a non-occupational injury or illness identified in our review underscore the need for RTW policies and programs that are sensitive to race and ethnicity. Importantly, our study calls for greater attention to the measurement of race and ethnicity in RTW and the generation of an evidence base that can inform the design of equitable work disability management practices.

## Electronic Supplementary Material

Below is the link to the electronic supplementary material.

Supplementary Material 1

Supplementary Material 2
