# Supplementary material for: Racial and Ethnic Inequities in the Return-to-Work of Workers Experiencing Injury or Illness: A Systematic Review
Source: J Occup Rehabil. 2023 Jun 9;33(3):432–49. doi: 10.1007/s10926-023-10119-1 (PMC10495511; doi:10.1007/s10926-023-10119-1)
Supplement: Supplementary file 1 — Supplementary Material 1 [file 10926_2023_10119_MOESM1_ESM.docx]

**Supplement 1: Data-base specific search terms**

*Database 1: Embase (Ovid)*

Database: Embase Classic+Embase <1947 to 2021 April 05>

Search Strategy:

--------------------------------------------------------------------------------

1     (Worker? or labo?rer?).ti,ab.

2     employee?.ti,ab.

3     employer?.ti,ab.

4     employment/

5     employment.ti,ab.

6     job?.ti,ab.

7     occupation*.ti,ab.

8     work/

9     workplace/

10     worksite*.ti,ab.

11     "work site*".ti,ab.

12     or/1-11

13     absenteeism/

14     absenteeism.ti,ab.

15     occupational disease/

16     (occupational adj2 disease*).ti,ab.

17     (occupational adj2 rehab*).ti,ab.

18     exp occupational accident/

19     (occupational adj2 injur*).ti,ab.

20     (occupational adj2 illness*).ti,ab.

21     (non-occupational adj2 illness*).ti,ab.

22     exp presenteeism/

23     presenteeism.ti,ab.

24     (work adj2 disabilit*).ti,ab.

25     (work* adj2 injur*).ti,ab.

26     exp workman compensation/

27     (worker* adj2 compensation).ti,ab.

28     (work* adj2 illness*).ti,ab.

29     (acquired adj2 condition*).ti,ab.

30     injur*.ti,ab.

31     illness*.ti,ab.

32     claimant*.ti,ab.

33     "short-term disabilit*".ti,ab.

34     "shortterm disabilit*".ti,ab.

35     "long-term disabilit*".ti,ab.

36     "longterm disabilit*".ti,ab.

37     (insurance adj2 claim*).ti,ab.

38     "job disruption".ti,ab.

39     or/13-38

40     chronic disease/

41     (chronic adj2 disease*).ti,ab.

42     disabilit*.ti,ab.

43     impairment.ti,ab.

44     "heart disease".ti,ab.

45     heart disease/

46     disabled person/

47     mental disease/

48     cardiovascular disease/

49     cardiovascular disease*.ti,ab.

50     cerebrovascular accident/

51     stroke.ti,ab.

52     amputee/

53     amputee?.ti,ab.

54     (mental adj2 illness*).ti,ab.

55     hearing impairment/

56     (hearing adj2 impair*).ti,ab.

57     visual disorder/

58     (visual adj2 impair*).ti,ab.

59     spinal cord injury/

60     spinal cord injur*.ti,ab.

61     traumatic brain injury/

62     traumatic brain injur*.ti,ab.

63     TBI.ti,ab.

64     malignant neoplasm/

65     cancer.ti,ab.

66     low back pain/

67     low back pain.ti,ab.

68     chronic pain/

69     (chronic adj2 pain).ti,ab.

70     injury/

71     trauma.ti,ab.

72     anxiety/

73     anxiety.ti,ab.

74     depression/

75     depression.ti,ab.

76     posttraumatic stress disorder/

77     post-traumatic stress.ti,ab.

78     posttraumatic stress.ti,ab.

79     (PTSD or PTSS).ti,ab.

80     exp workplace violence/

81     (workplace adj2 violence).ti,ab.

82     bullying/

83     bullying.ti,ab.

84     (disease adj2 flare*).ti,ab.

85     (episodic adj2 disabilit*).ti,ab.

86     fatigue/)

87     fatigue.ti,ab.

88     rheumatic disease/

89     (rheumatic adj2 disease*).ti,ab.

90     or/40-89

91     12 and 39

92     12 and 90

93     91 or 92

94     African American/

95     African Continental Ancestry Group/

96     Asian continental ancestry group/

97     American Native Continental Ancestry Group/

98     Middle East/ or North Africa Ancestry Group/

99     Caucasian/

100     European Continental Ancestry Group/

101     cross-cultural comparison/

102     exp Ethnic groups/

103     Oceanic Ancestry Group/

104     "African American*".ti,ab.

105     Asian*.ti,ab.

106     Black.ti,ab.

107     Blacks.ti,ab.

108     Caucasian.ti,ab.

109     cross cultural.ti,ab.

110     ethnic.ti,ab.

111     ethnicity.ti,ab.

112     hispanic*.ti,ab.

113     Latina*.ti,ab.

114     Latino*.ti,ab.

115     Native American*.ti,ab.

116     race.ti,ab.

117     races.ti,ab.

118     racial.ti,ab.

119     white.ti,ab.

120     whites.ti,ab.

121     Caribbean.ti,ab.

122     Indigenous.ti,ab.

123     Aboriginal$.ti,ab.

124     Metis.ti,ab.

125     Inuit.ti,ab.

126     Maori$.ti,ab.

127     First Nation$.ti,ab.

128     First People$.ti,ab.

129     American Indian$.ti,ab.

130     Amerindian*.ti,ab.

131     Eskimo$.ti,ab.

132     Native Canadian$.ti,ab.

133     Native Hawaiian$.ti,ab.

134     Native People$.ti,ab.

135     Native population.ti,ab.

136     (tribal or tribe).ti,ab.

137     North American Native.ti,ab.

138     (Saami or Sami).ti,ab.

139     visible minorit*.ti,ab.

140     non-white.ti,ab.

141     Indian$.ti,ab.

142     (India or India's).ti,ab. or India/

143     141 not 142

144     or/94-140

145     143 or 144

146     discrimin*.ti,ab.

147     bias.ti,ab.

148     prejud*.ti,ab.

149     hostil*.ti,ab.

150     harass*.ti,ab.

151     bully*.ti,ab.

152     "unfair treat*".ti,ab.

153     oppress*.ti,ab.

154     exp prejudice/

155     racis*.ti,ab.

156     ethnic*.ti,ab.

157     cultur*.ti,ab.

158     religio*.ti,ab.

159     (migrant* or migration).ti,ab.

160     religio*.ti,ab.

161     refugee*.ti,ab.

162     exp minority group/

163     immigra*.ti,ab.

164     "Emigrants and Immigrants"/

165     Undocumented immigrants/

166     (racial adj2 (disparity or disparities or inequalities or inequities)).ti,ab.

167     segregation.ti,ab.

168     or/146-167

169     145 or 168

170     return to work/

171     absenteeism/

172     absenteeism.ti,ab.

173     alternative work$.ti,ab.

174     alternative task?.ti,ab.

175     (attendance adj2 work).ti,ab.

176     (benefit? adj2 duration).ti,ab.

177     (communicat$ adj2 employer?).ti,ab.

178     (communicat$ adj2 "health care").ti,ab.

179     (communicat$ adj2 healthcare).ti,ab.

180     (communicat$ adj2 "work place?").ti,ab.

181     (communicat$ adj2 workplace?).ti,ab.

182     compensation claim? cost?.ti,ab.

183     (claim$ adj2 cost?).ti,ab.

184     compensation cost?.ti,ab.

185     continuance cost?.ti,ab.

186     continuance rate?.ti,ab.

187     fit note?.ti,ab.

188     functional limitation?.ti,ab.

189     graded activit$.ti,ab.

190     Health/

191     health-related work role functioning.ti,ab.

192     Health Status/

193     (injur$ adj2 experience?).ti,ab.

194     income replacement.ti,ab.

195     labo?r market re-entry.ti,ab.

196     labo?r market reentry.ti,ab.

197     liability reduction.ti,ab.

198     long-term disabilit$.ti,ab.

199     longterm disabilit$.ti,ab.

200     long-term insurance.ti,ab.

201     lost time.ti,ab.

202     (lost adj2 "work day?").ti,ab.

203     (lost adj2 workday?).ti,ab.

204     (maintenance adj2 work).ti,ab.

205     physical capacity.ti,ab.

206     precarious work$ arrangement?.ti,ab.

207     presenteeism.ti,ab.

208     re-employ$.ti,ab.

209     reemploy$.ti,ab.

210     re-injur$.ti,ab.

211     reinjur$.ti,ab.

212     reasonable accommodation?.ti,ab.

213     recurrent disease/

214     (redeployed adj2 job).ti,ab.

215     relapse?.ti,ab.

216     (return$ adj3 work$).ti,ab.

217     second employer program$.ti,ab.

218     second$ injur$.ti,ab.

219     short term disabilit$.ti,ab.

220     short-term disabilit$.ti,ab.

221     medical leave/

222     sick list$.ti,ab.

223     (sick$ adj2 absence?).ti,ab.

224     social exclusion.ti,ab.

225     suitable duties.ti,ab.

226     suitable duty.ti,ab.

227     suitable employment.ti,ab.

228     (support$ adj2 co-worker?).ti,ab.

229     (support$ adj2 coworker?).ti,ab.

230     (support$ adj2 colleague?).ti,ab.

231     (support$ adj2 manager?).ti,ab.

232     (support$ adj2 supervisor?).ti,ab.

233     sustainable employment.ti,ab.

234     time loss.ti,ab.

235     time lost.ti,ab.

236     (time adj1 benefit?).ti,ab.

237     wage replace$.ti,ab.

238     (work$ adj2 accommodat$).ti,ab.

239     work ability.ti,ab.

240     workability.ti,ab.

241     work capacity.ti,ab.

242     (work$ adj2 disab$).ti,ab.

243     (work$ adj2 limit$).ti,ab.

244     (work$ adj2 maintenance).ti,ab.

245     (work$ adj2 participation).ti,ab.

246     (work$ adj2 re-integrat$).ti,ab.

247     (work$ adj2 reintegrat$).ti,ab.

248     work readiness.ti,ab.

249     work role function$.ti,ab.

250     worklessness.ti,ab.

251     alternat$ duty.ti,ab.

252     alternat$ duties.ti,ab.

253     case management/

254     disability management program$.ti,ab.

255     Flexible work$.ti,ab.

256     (modifi$ adj2 duties).ti,ab.

257     (modifi$ adj2 duty).ti,ab.

258     (modifi$ adj2 work).ti,ab.

259     (longterm adj2 "income loss*").ti,ab.

260     (long-term adj2 "income loss*").ti,ab.

261     financial hardship*.ti,ab.

262     financial stress*.ti,ab.

263     poverty.ti,ab.

264     poverty/

265     ("employment opportunit*" adj2 loss*).ti,ab.

266     (productivity adj2 loss*).ti,ab.

267     "future earning capacity".ti,ab.

268     ("career path" adj2 damag*).ti,ab.

269     or/170-268

270     93 and 169 and 269

271     limit 270 to yr=2001-2021

*Database 2: MEDLINE (Ovid)*

Ovid MEDLINE: Epub Ahead of Print, In-Process & Other Non-Indexed Citations, Ovid MEDLINE® Daily and Ovid MEDLINE® <1946-Present>

Search Strategy:

--------------------------------------------------------------------------------

1     (Worker? or labo?rer?).ti,ab.

2     employee?.ti,ab.

3     employer?.ti,ab.

4     Employment/

5     employment.ti,ab.

6     job?.ti,ab.

7     occupation*.ti,ab.

8     work/

9     workplace/

10     worksite*.ti,ab.

11     "work site*".ti,ab.

12     or/1-11

13     absenteeism/

14     absenteeism.ti,ab.

15     Occupational Diseases/

16     (occupational adj2 disease*).ti,ab.

17     (occupational adj2 rehab*).ti,ab.

18     Occupational Injuries/

19     (occupational adj2 injur*).ti,ab.

20     (occupational adj2 illness*).ti,ab.

21     (non-occupational adj2 illness*).ti,ab.

22     exp Presenteeism/

23     presenteeism.ti,ab.

24     (work adj2 disabilit*).ti,ab.

25     (work* adj2 injur*).ti,ab.

26     exp Workers' Compensation/

27     (worker* adj2 compensation).ti,ab.

28     (work* adj2 illness*).ti,ab.

29     (acquired adj2 condition*).ti,ab.

30     injur*.ti,ab.

31     illness*.ti,ab.

32     claimant*.ti,ab.

33     "short-term disabilit*".ti,ab.

34     "shortterm disabilit*".ti,ab.

35     "long-term disabilit*".ti,ab.

36     "longterm disabilit*".ti,ab.

37     (insurance adj2 claim*).ti,ab.

38     "job disruption".ti,ab.

39     or/13-38

40     Chronic Disease/

41     (chronic adj2 disease*).ti,ab.

42     disabilit*.ti,ab.

43     impairment.ti,ab.

44     "heart disease".ti,ab.

45     Heart Diseases/

46     Disabled Persons/

47     mental disorders/

48     Cardiovascular Diseases/

49     cardiovascular disease*.ti,ab.

50     Stroke/

51     stroke.ti,ab.

52     Amputees/

53     amputee?.ti,ab.

54     (mental adj2 illness*).ti,ab.

55     Hearing Loss/

56     (hearing adj2 impair*).ti,ab.

57     Vision Disorders/

58     (visual* adj2 impair*).ti,ab.

59     Spinal Cord Injuries/

60     spinal cord injur*.ti,ab.

61     Brain Injuries, Traumatic/

62     traumatic brain injur*.ti,ab.

63     TBI.ti,ab.

64     Neoplasms/

65     cancer.ti,ab.

66     Low Back Pain/

67     low back pain.ti,ab.

68     Chronic Pain/

69     (chronic adj2 pain).ti,ab.

70     "Wounds and Injuries"/

71     trauma.ti,ab.

72     Anxiety/

73     anxiety.ti,ab.

74     Depression/

75     depression.ti,ab.

76     Stress Disorders, Post-Traumatic/

77     stress disorders, traumatic/

78     post-traumatic stress.ti,ab.

79     posttraumatic stress.ti,ab.

80     (PTSD or PTSS).ti,ab.

81     exp Workplace Violence/

82     (workplace* adj2 violence).ti,ab.

83     Bullying/

84     bullying.ti,ab.

85     (disease adj2 flare*).ti,ab.

86     (episodic adj2 disabilit*).ti,ab.

87     Fatigue/

88     fatigue.ti,ab.

89     Rheumatic Diseases/

90     (rheumatic adj2 disease*).ti,ab.

91     or/40-90

92     12 and 39

93     12 and 91

94     92 or 93

95     African Continental Ancestry Group/

96     Asian Continental Ancestry Group/

97     American Native Continental Ancestry Group/

98     Middle East/ or North Africa Ancestry Group/

99     European Continental Ancestry Group/

100     cross-cultural comparison/

101     exp Ethnic Groups/

102     Oceanic Ancestry Group/

103     "African American*".ti,ab.

104     Asian*.ti,ab.

105     black.ti,ab.

106     Blacks.ti,ab.

107     Caucasian*.ti,ab.

108     cross cultural.ti,ab.

109     ethnic.ti,ab.

110     ethnicity.ti,ab.

111     hispanic*.ti,ab.

112     Latina*.ti,ab.

113     Latino*.ti,ab.

114     Native American*.ti,ab.

115     race.ti,ab.

116     races.ti,ab.

117     racial.ti,ab.

118     white.ti,ab.

119     whites.ti,ab.

120     Caribbean.ti,ab.

121     Indigenous.ti,ab.

122     Aboriginal$.ti,ab.

123     Metis.ti,ab.

124     Inuit.ti,ab.

125     Maori$.ti,ab.

126     First Nation$.ti,ab.

127     First People$.ti,ab.

128     American Indian$.ti,ab.

129     Amerindian*.ti,ab.

130     Eskimo$.ti,ab.

131     Native Canadian$.ti,ab.

132     Native Hawaiian$.ti,ab.

133     Native peoples$.ti,ab.

134     Native population.ti,ab.

135     (tribal or tribe).ti,ab.

136     North American Native.ti,ab.

137     (Saami or Sami).ti,ab.

138     visible minorit*.ti,ab.

139     non-white.ti,ab.

140     Indian$.ti,ab.

141     (India or India's).ti,ab. or India/

142     140 not 141

143     or/95-139

144     142 or 143

145     racis*.ti,ab.

146     discrim*.ti,ab.

147     bias.ti,ab.

148     prejud*.ti,ab.

149     hostil*.ti,ab.

150     harass*.ti,ab.

151     bully*.ti,ab.

152     "unfair treat*".ti,ab.

153     oppress*.ti,ab.

154     exp Prejudice/

155     ethnic*.ti,ab.

156     cultur*.ti,ab.

157     religio*.ti,ab.

158     (migrant* or migration).ti,ab.

159     refugee*.ti,ab.

160     exp Minority Groups/

161     immigra*.ti,ab.

162     "Emigrants and Immigrants"/

163     Undocumented Immigrants/

164     (racial adj2 (disparity or disparities or inequalities or inequities)).ti,ab.

165     segregation.ti,ab.

166     or/145-165

167     144 or 166

168     Return to Work/

169     Absenteeism/

170     absenteeism.ti,ab.

171     alternative work$.ti,ab.

172     alternative task?.ti,ab.

173     (attendance adj2 work).ti,ab.

174     (benefit? adj2 duration).ti,ab.

175     (communicat$ adj2 employer?).ti,ab.

176     (communicat$ adj2 "health care").ti,ab.

177     (communicat$ adj2 healthcare).ti,ab.

178     (communicat$ adj2 "work place?").ti,ab.

179     (communicat$ adj2 workplace?).ti,ab.

180     compensation claim? cost?.ti,ab.

181     (claim$ adj2 cost?).ti,ab.

182     compensation cost?.ti,ab.

183     continuance cost?.ti,ab.

184     continuance rate?.ti,ab.

185     fit note?.ti,ab.

186     functional limitation?.ti,ab.

187     graded activit$.ti,ab.

188     health/

189     health-related work role functioning.ti,ab.

190     Health Status/

191     (injur$ adj2 experience?).ti,ab.

192     income replacement.ti,ab.

193     labo?r market re-entry.ti,ab.

194     labo?r market reentry.ti,ab.

195     liability reduction.ti,ab.

196     long-term disabilit$.ti,ab.

197     longterm disabilit$.ti,ab.

198     long-term insurance.ti,ab.

199     lost time.ti,ab.

200     (lost adj2 "work day?").ti,ab.

201     (lost adj2 workday?).ti,ab. (366)

202     (maintenance adj2 work).ti,ab.

203     physical capacity.ti,ab.

204     precarious work$ arrangement?.ti,ab.

205     presenteeism.ti,ab.

206     re-employ$.ti,ab.

207     reemploy$.ti,ab.

208     re-injur$.ti,ab.

209     reinjur$.ti,ab.

210     reasonable accommodation?.ti,ab.

211     Recurrence/

212     (redeployed adj2 job).ti,ab.

213     relapse?.ti,ab.

214     (return$ adj3 work$).ti,ab.

215     second employer program$.ti,ab.

216     second$ injur$.ti,ab.

217     short term disabilit$.ti,ab.

218     short-term disabilit$.ti,ab.

219     Sick Leave/

220     sick list$.ti,ab.

221     (sick$ adj2 absence?).ti,ab.

222     social exclusion.ti,ab.

223     suitable duties.ti,ab.

224     suitable duty.ti,ab.

225     suitable employment.ti,ab.

226     (support$ adj2 co-worker?).ti,ab.

227     (support$ adj2 coworker?).ti,ab.

228     (support$ adj2 colleague?).ti,ab.

229     (support$ adj2 manager?).ti,ab.

230     (support$ adj2 supervisor?).ti,ab.

231     sustainable employment.ti,ab.

232     time loss.ti,ab.

233     time lost.ti,ab.

234     (time adj1 benefit?).ti,ab.

235     wage replace$.ti,ab.

236     (work$ adj2 accommodat$).ti,ab.

237     work ability.ti,ab.

238     workability.ti,ab.

239     work capacity.ti,ab.

240     (work$ adj2 disab$).ti,ab.

241     (work$ adj2 limit$).ti,ab.

242     (work$ adj2 maintenance).ti,ab.

243     (work$ adj2 participation).ti,ab.

244     (work$ adj2 re-integrat$).ti,ab.

245     (work$ adj2 reintegrat$).ti,ab.

246     work readiness.ti,ab.

247     work role function$.ti,ab.

248     worklessness.ti,ab.

249     alternat$ duty.ti,ab.

250     alternat$ duties.ti,ab.

251     Case Management/

252     disability management program$.ti,ab.

253     Flexible work$.ti,ab.

254     (modifi$ adj2 duties).ti,ab.

255     (modifi$ adj2 duty).ti,ab.

256     (modifi$ adj2 work).ti,ab.

257     (longterm adj2 "income loss*").ti,ab.

258     (long-term adj2 "income loss*").ti,ab.

259     financial hardship*.ti,ab.

260     financial stress*.ti,ab.

261     poverty.ti,ab.

262     Poverty/

263     ("employment opportunit*" adj2 loss*).ti,ab.

264     (productivity adj2 loss*).ti,ab.

265     "future earning capacity".ti,ab.

266     ("career path" adj2 damag*).ti,ab.

267     or/168-266

268     94 and 167 and 267

269     limit 268 to yr=2001-2021

*Database 3: APA PsycINFO (Ovid)*

Database: APA PsycInfo <1806 to March Week 5 2021>

Search Strategy:

--------------------------------------------------------------------------------

1     (Worker? or labo?rer?).ti,ab.

2     employee?.ti,ab.

3     employer?.ti,ab.

4     Employment Status/

5     employment.ti,ab.

6     job?.ti,ab.

7     occupation*.ti,ab.

8     workplace*.ti,ab.

9     worksite*.ti,ab.

10     "work site*".ti,ab.

11     or/1-10

12     Employee Absenteeism/

13     absenteeism.ti,ab.

14     exp Occupational Health/

15     (occupational adj2 disease*).ti,ab.

16     (occupational adj2 rehab*).ti,ab.

17     exp Occupational Safety/

18     (occupational adj2 injur*).ti,ab.

19     (occupational adj2 illness*).ti,ab.

20     (non-occupational adj2 illness*).ti,ab.

21     presenteeism.ti,ab.

22     (work adj2 disabilit*).ti,ab.

23     (work* adj2 injur*).ti,ab.

24     exp Workers' Compensation Insurance/

25     (worker* adj2 compensation).ti,ab.

26     (work* adj2 illness*).ti,ab.

27     (acquired adj2 condition*).ti,ab.

28     injur*.ti,ab.

29     illness*.ti,ab.

30     claimant*.ti,ab.

31     "short-term disabilit*".ti,ab.

32     "shortterm disabilit*".ti,ab.

33     "long-term disabilit*".ti,ab.

34     "longterm disabilit*".ti,ab.

35     (insurance adj2 claim*).ti,ab.

36     "job disruption".ti,ab.

37     or/12-36

38     Chronic Illness/

39     (chronic adj2 disease*).ti,ab.

40     disabilit*.ti,ab.

41     impairment.ti,ab.

42     "heart disease".ti,ab.

43     Heart Disorders/

44     Disabilities/

45     Mental Disorders/

46     Cardiovascular Disorders/

47     cardiovascular disease*.ti,ab.

48     Cerebrovascular Accidents/

49     stroke.ti,ab.

50     Amputation/

51     amputee?.ti,ab.

52     (mental adj2 illness*).ti,ab.

53     Hearing Disorders/

54     (hearing adj2 impair*).ti,ab.

55     Vision Disorders/

56     (visual* adj2 impair*).ti,ab.

57     Spinal Cord Injuries/

58     spinal cord injur*.ti,ab.

59     Traumatic Brain Injury/

60     traumatic brain injur*.ti,ab.

61     TBI.ti,ab.

62     Neoplasms/

63     cancer.ti,ab.

64     Back Pain/

65     low back pain.ti,ab.

66     Chronic Pain/

67     (chronic adj pain).ti,ab.

68     exp Trauma/

69     trauma.ti,ab.

70     Anxiety/

71     anxiety.ti,ab.

72     Major Depression/

73     depression.ti,ab.

74     Posttraumatic Stress Disorder/

75     post-traumatic stress.ti,ab.

76     posttraumatic stress.ti,ab.

77     (PTSD or PTSS).ti,ab.

78     Workplace Violence/

79     (workplace* adj2 violence).ti,ab.

80     Bullying/

81     bullying.ti,ab.

82     (disease adj2 flare*).ti,ab.

83     (episodic adj2 disabilit*).ti,ab.

84     Fatigue/

85     fatigue.ti,ab.

86     Rheumatoid Arthritis/

87     (rheumatic adj2 disease*).ti,ab.

88     or/38-87

89     11 and 37

90     11 and 88

91     89 or 90

92     African Cultural Groups/

93     Asians/

94     American Indians/

95     Whites/

96     Cross Cultural Differences/

97     exp "Racial and Ethnic Groups"/

98     "African American*".ti,ab.

99     Asian*.ti,ab.

100     black.ti,ab.

101     Blacks.ti,ab.

102     Caucasian*.ti,ab.

103     cross cultural.ti,ab.

104     ethnic.ti,ab.

105     ethnicity.ti,ab.

106     hispanic*.ti,ab.

107     Latina*.ti,ab.

108     Latino*.ti,ab.

109     Native American*.ti,ab.

110     race.ti,ab.

111     races.ti,ab.

112     racial.ti,ab.

113     white.ti,ab.

114     whites.ti,ab.

115     Caribbean.ti,ab.

116     Indigenous.ti,ab.

117     Aboriginal$.ti,ab.

118     Metis.ti,ab.

119     Inuit.ti,ab.

120     Maori$.ti,ab.

121     First Nation$.ti,ab.

122     First People$.ti,ab.

123     American Indian$.ti,ab.

124     Amerindian*.ti,ab.

125     Eskimo$.ti,ab.

126     Native Canadian$.ti,ab.

127     Native Hawaiian$.ti,ab.

128     Native peoples$.ti,ab.

129     Native population.ti,ab.

130     (tribal or tribe).ti,ab.

131     North American Native.ti,ab.

132     (Saami or Sami).ti,ab.

133     visible minorit*.ti,ab.

134     non-white.ti,ab.

135     Indian$.ti,ab.

136     (India or India's).ti,ab. or India/

137     135 not 136

138     or/92-135

139     137 or 138

140     racis*.ti,ab.

141     discrim*.ti,ab.

142     bias.ti,ab.

143     prejud*.ti,ab.

144     hostil*.ti,ab.

145     harass*.ti,ab.

146     bully*.ti,ab.

147     "unfair treat*".ti,ab.

148     oppress*.ti,ab.

149     exp Prejudice/

150     ethnic*.ti,ab.

151     cultur*.ti,ab.

152     religio*.ti,ab.

153     (migrant* or migration).ti,ab.

154     refugee*.ti,ab.

155     exp Minority Groups/

156     Immigration/

157     immigra*.ti,ab.

158     (racial adj2 (disparity or disparities or inequalities or inequities)).ti,ab.

159     segregation.ti,ab.

160     or/140-159

161     139 or 160

162     EMPLOYEE ABSENTEEISM/

163     absenteeism.ti,ab.

164     alternative work$.ti,ab.

165     alternative task?.ti,ab.

166     (attendance adj2 work).ti,ab.

167     (benefit? adj2 duration).ti,ab.

168     (communicat$ adj2 employer?).ti,ab.

169     (communicat$ adj2 "health care").ti,ab.

170     (communicat$ adj2 healthcare).ti,ab.

171     (communicat$ adj2 "work place?").ti,ab.

172     (communicat$ adj2 workplace?).ti,ab.

173     compensation claim? cost?.ti,ab.

174     (claim$ adj2 cost?).ti,ab.

175     compensation cost?.ti,ab.

176     continuance cost?.ti,ab.

177     continuance rate?.ti,ab.

178     fit note?.ti,ab.

179     functional limitation?.ti,ab.

180     graded activit$.ti,ab.

181     HEALTH/

182     health-related work role functioning.ti,ab.

183     Health Status/

184     (injur$ adj2 experience?).ti,ab.

185     income replacement.ti,ab.

186     labo?r market re-entry.ti,ab.

187     labo?r market reentry.ti,ab.

188     liability reduction.ti,ab.

189     long-term disabilit$.ti,ab.

190     longterm disabilit$.ti,ab.

191     long-term insurance.ti,ab.

192     lost time.ti,ab.

193     (lost adj2 "work day?").ti,ab.

194     (lost adj2 workday?).ti,ab.

195     (maintenance adj2 work).ti,ab.

196     physical capacity.ti,ab.

197     precarious work$ arrangement?.ti,ab.

198     presenteeism.ti,ab.

199     re-employ$.ti,ab.

200     reemploy$.ti,ab.

201     re-injur$.ti,ab.

202     reinjur$.ti,ab.

203     reasonable accommodation?.ti,ab.

204     "Relapse (Disorders)"/

205     (redeployed adj2 job).ti,ab.

206     relapse?.ti,ab.

207     Reemployment/

208     (return$ adj3 work$).ti,ab.

209     second employer program$.ti,ab.

210     second$ injur$.ti,ab.

211     short term disabilit$.ti,ab.

212     short-term disabilit$.ti,ab.

213     Employee Leave Benefits/

214     sick list$.ti,ab.

215     (sick$ adj2 absence?).ti,ab.

216     social exclusion.ti,ab.

217     suitable duties.ti,ab.

218     suitable duty.ti,ab.

219     suitable employment.ti,ab.

220     (support$ adj2 co-worker?).ti,ab.

221     (support$ adj2 coworker?).ti,ab.

222     (support$ adj2 colleague?).ti,ab.

223     (support$ adj2 manager?).ti,ab.

224     (support$ adj2 supervisor?).ti,ab.

225     sustainable employment.ti,ab.

226     time loss.ti,ab.

227     time lost.ti,ab.

228     (time adj1 benefit?).ti,ab.

229     wage replace$.ti,ab.

230     (work$ adj2 accommodat$).ti,ab.

231     work ability.ti,ab.

232     workability.ti,ab.

233     work capacity.ti,ab.

234     (work$ adj2 disab$).ti,ab.

235     (work$ adj2 limit$).ti,ab.

236     (work$ adj2 maintenance).ti,ab.

237     (work$ adj2 participation).ti,ab.

238     (work$ adj2 re-integrat$).ti,ab.

239     (work$ adj2 reintegrat$).ti,ab.

240     work readiness.ti,ab.

241     work role function$.ti,ab.

242     worklessness.ti,ab.

243     alternat$ duty.ti,ab.

244     alternat$ duties.ti,ab.

245     Case Management/

246     disability management program$.ti,ab.

247     Flexible work$.ti,ab.

248     (modifi$ adj2 duties).ti,ab.

249     (modifi$ adj2 duty).ti,ab.

250     (modifi$ adj2 work).ti,ab.

251     (longterm adj2 "income loss*").ti,ab.

252     (long-term adj2 "income loss*").ti,ab.

253     financial hardship*.ti,ab.

254     financial stress*.ti,ab.

255     poverty.ti,ab.

256     Poverty/

257     ("employment opportunit*" adj2 loss*).ti,ab.

258     (productivity adj2 loss*).ti,ab.

259     "future earning capacity".ti,ab.

260     ("career path" adj2 damag*).ti,ab.

261     or/162-260

262     91 and 161 and 261

263     limit 262 to yr=2001-2021

*Database 4: CINAHL (EBSCO)*

S267 S266 Limiters - Published Date: 20010101-20210431; Peer Reviewed; Language: English, French

S266 S265 Limiters - Published Date: 20010101-20210431

S265 S93 AND S164 AND S264

S264 S164 OR S165 OR S166 OR S167 OR S168 OR S169 OR S170 OR S171 OR S172 OR S173 OR S174 OR S175 OR S176 OR S177 OR S178 OR S179 OR S180 OR S181 OR S182 OR S183 OR S184 OR S185 OR S186 OR S187 OR S188 OR S189 OR S190 OR S191 OR S192 OR S193 OR S194 OR S195 OR S196 OR S197 OR S198 OR S199 OR S200 OR S201 OR S202 OR S203 OR S204 OR S205 OR S206 OR S207 OR S208 OR S209 OR S210 OR S211 OR S212 OR S213 OR S214 OR S215 OR S216 OR S217 OR S218 OR S219 OR S220 OR S221 OR S222 OR S223 OR S224 OR S225 OR S226 OR S227 OR S228 OR S229 OR S230 OR S231 OR S232 OR S233 OR S234 OR S235 OR S236 OR S237 OR S238 OR S239 OR S240 OR S241 OR S242 OR S243 OR S244 OR S245 OR S246 OR S247 OR S248 OR S249 OR S250 OR S251 OR S252 OR S253 OR S254 OR S255 OR S256 OR S257 OR S258 OR S259 OR S260 OR S261 OR S262 OR S263

S263 TI ("career path" N2 damag*) OR AB ("career path" N2 damag*)

S262 TI "future earning capacity" OR AB "future earning capacity"

S261 TI (productivity N2 loss*) OR AB (productivity N2 loss*)

S260 TI ("employment opportunit*" N2 loss*) OR AB ("employment opportunit*" N2 loss*)

S259 (MH "Poverty")

S258 TI poverty OR AB poverty

S257 TI financial stress* OR AB financial stress*

S256 TI financial hardship* OR AB financial hardship*

S255 TI (long-term N2 "income loss*") OR AB (long-term N2 "income loss*")

S254 TI (longterm N2 "income loss*") OR AB (longterm N2 "income loss*")

S253 TI (modifi* N2 work) OR AB (modifi* N2 work)

S252 TI (modifi* N2 duties) OR AB (modifi* N2 duties)

S251 TI (modifi* N2 duty) OR AB (modifi* N2 duty)

S250 TI Flexible work* OR AB Flexible work*

S249 TI disability management program* OR AB disability management program*

S248 (MH "Case Management")

S247 TI alternat* duties OR AB alternat* duties

S246 TI alternat* duty OR AB alternat* duty

S245 TI worklessness OR AB worklessness

S244 TI work role function* OR AB work role function*

S243 TI work readiness OR AB work readiness

S242 TI (work* N2 reintegrat*) OR AB work* N2 reintegrat*)

S241 TI (work* N2 re-integrat*) OR AB work* N2 re-integrat*)

S240 TI (work* N2 participation) OR AB (work* N2 participation)

S239 TI (work* N2 maintenance) OR AB (work* N2 maintenance)

S238 TI (work* N2 limit*) OR AB (work* N2 limit*)

S237 TI (work* N2 disab*) OR AB (work* N2 disab*)

S236 TI work capacity OR AB work capacity

S235 TI workability OR AB workability

S234 TI work ability OR AB work ability

S233 TI (work* N2 accommodat*) OR AB (work* N2 accommodat*)

S232 TI wage replace* OR AB wage replace*

S231 TI (time N1 benefit#) OR AB (time N1 benefit#)

S230 TI time lost OR AB time lost

S229 TI time loss OR AB time loss

S228 TI sustainable employment OR AB sustainable employment

S227 TI (support* N2 supervisor#) OR AB (support* N2 supervisor#)

S226 TI (support* N2 manager#) OR AB (support* N2 manager#)

S225 TI (support* N2 colleague#) OR AB (support* N2 colleague#)

S224 TI (support* N2 coworker#) OR AB (support* N2 coworker#)

S223 TI (support* N2 co-worker#) OR AB (support* N2 co-worker#)

S222 TI suitable employment OR AB suitable employment

S221 TI suitable duty OR AB suitable duty

S220 TI suitable duties OR AB suitable duties

S219 TI social exclusion OR AB social exclusion

S218 TI (sick* N2 absence#) OR AB (sick* N2 absence#)

S217 TI sick list# OR AB sick list#

S216 (MH "Sick Leave")

S215 TI short-term disabilit* OR AB short-term disabilit*

S214 TI short term disabilit* OR AB short term disabilit*

S213 TI second* injur* OR AB second* injur*

S212 TI second employer program# OR AB second employer program#

S211 TI (return* N2 work*) OR AB (return* N2 work*)

S210 TI relapse# OR AB relapse#

S209 TI (redeployed N2 job) OR AB (redeployed N2 job)

S208 MH “Recurrence”

S207 TI reasonable accommodation# OR AB reasonable accommodation#

S206 TI reinjur* OR AB reinjur*

S205 TI re-injur* OR AB re-injur*

S204 TI reemploy* OR AB reemploy*

S203 TI re-employ* OR AB re-employ*

S202 TI presenteeism OR AB presenteeism

S201 TI "precarious work* arrangement#" OR AB "precarious work* arrangement#"

S200 TI physical capacity OR AB physical capacity

S199 TI (maintenance N2 work) OR AB (maintenance N2 work)

S198 TI (lost N2 workday*) OR AB (lost N2 workday*)

S197 TI (lost N2 "work day*") OR AB (lost N2 "work day*")

S196 TI "lost time" OR AB "lost time"

S195 TI "long-term insurance" OR AB "long-term insurance"

S194 TI "longterm disabilit*" OR AB "longterm disabilit*"

S193 TI "long-term disabilit*" OR AB "long-term disabilit*"

S192 TI liability reduction OR AB liability reduction

S191 TI "labo#r market re-entry" OR AB "labo#r market re-entry"

S190 TI "labo#r market reentry" OR AB "labo#r market reentry"

S189 TI income replacement OR AB income replacement

S188 TI (injur# N2 experience#) OR AB (injur# N2 experience#)

S187 (MH "Health Status")

S186 TI health-related work role functioning OR AB health-related work role functioning

S185 (MH "Health")

S184 TI graded activit* OR AB graded activit*

S183 TI functional limitation# OR AB functional limitation#

S182 TI fit note# OR AB fit note#

S181 TI continuance rate# OR AB continuance rate#

S180 TI continuance cost# OR AB continuance cost#

S179 TI compensation cost# OR AB compensation cost#

S178 TI (claim* N2 cost#) OR AB (claim* N2 cost#)

S177 TI "compensation claim# cost#" OR AB "compensation claim# cost#"

S176 TI (communicat* N2 workplace#) OR AB (communicat* N2 workplace#)

S175 TI (communicat* N2 "work place#") OR AB (communicat* N2 "work place#")

S174 TI (communicat* N2 healthcare) OR AB (communicat* N2 healthcare)

S173 TI (communicat* N2 "health care") OR AB (communicat* N2 "health care")

S172 TI (communicat* N2 employer#) OR AB (communicat* N2 employer#)

S171 TI (benefit* N2 duration) OR AB (benefit* N2 duration)

S170 TI (attendance N2 work) OR AB (attendance N2 work)

S169 TI "alternative task*" OR AB "alternative task*"

S168 TI "alternative work*" OR AB "alternative work*"

S167 TI absenteeism OR AB absenteeism

S166 (MH "Absenteeism")

S165 (MH "Job Re-Entry")

S164 S142 OR S163

S163 S143 OR S144 OR S145 OR S146 OR S147 OR S148 OR S149 OR S150 OR S151 OR S152 OR S153 OR S154 OR S155 OR S156 OR S157 OR S158 OR S159 OR S160 OR S161 OR S162

S162 ( TI (racial N2 (disparity OR disparities OR inequalities OR inequities)) ) OR ( AB (racial N2 (disparity OR disparities OR inequalities OR inequities)) )

S161 (MH "Immigrants, Illegal")

S160 (MH "Immigrants")

S159 TI immigra* OR AB immigra*

S158 (MH "Minority Groups")

S157 TI refugee* OR AB refugee*

S156 TI (migrant# or migration) OR AB (migrant# or migration)

S155 TI religio* OR AB religio*

S154 TI cultur* OR AB cultur*

S153 TI ethnic* OR AB ethnic*

S152 (MH "Prejudice+")

S151 TI oppress* OR AB oppress*

S150 TI "unfair treat*" OR AB "unfair treat*"

S149 TI bully* OR AB bully*

S148 TI harass* OR AB harass*

S147 TI hostil* OR AB hostil*

S146 TI prejud* OR AB prejud*

S145 TI bias OR AB bias

S144 TI discrimin* OR AB discrimin*

S143 TI racis* OR AB racis*

S142 S138 OR S141

S141 S139 NOT S140

S140 (MH "India") OR ( TI India OR AB India ) OR ( TI India's OR AB India's )

S139 TI Indian# OR AB Indian#

S138 S94 OR S95 OR S96 OR S97 OR S98 OR S99 OR S100 OR S101 OR S102 OR S103 OR S104 OR S105 OR S106 OR S107 OR S108 OR S109 OR S110 OR S111 OR S112 OR S113 OR S114 OR S115 OR S116 OR S117 OR S118 OR S119 OR S120 OR S121 OR S122 OR S123 OR S124 OR S125 OR S126 OR S127 OR S128 OR S129 OR S130 OR S131 OR S132 OR S133 OR S134 OR S135 OR S136 OR S137 S137

TI non-white OR AB non-white

S136 TI "visible minorit*" OR AB "visible minorit*"

S135 TI (Saami or Sami) OR AB (Saami or Sami)

S134 TI "North American native#" OR AB "North American native#"

S133 TI (tribal or tribe#) OR AB (tribal or tribe#)

S132 TI "Native population" OR AB "Native population"

S131 TI "Native people#" OR AB "Native people#"

S130 TI "Native Hawaiian*" OR AB "Native Hawaiian*"

S129 TI "Native Canadian*" OR AB "Native Canadian*"

S128 TI Eskimo# OR AB Eskimo#

S127 TI Amerindian# OR AB Amerindian#

S126 TI "American Indian*" OR AB "American Indian*"

S125 TI "First People#" OR AB "First People#"

S124 TI "First Nation#" OR AB "First Nation#"

S123 TI Maori* OR AB Maori*

S122 TI Inuit OR AB Inuit

S121 TI Metis OR AB Metis

S120 TI Aboriginal* OR AB Aboriginal*

S119 TI Indigenous OR AB Indigenous

S118 TI Caribbean OR AB Caribbean

S117 TI whites OR AB whites

S116 TI white OR AB white

S115 TI racial OR AB racial

S114 TI races OR AB races

S113 TI race OR AB race

S112 TI "Native American*" OR AB "Native American*"

S111 TI Latino* OR AB Latino*

S110 TI Latina* OR AB Latina*

S109 TI hispanic* OR AB hispanic*

S108 TI ethnicity OR AB ethnicity

S107 TI ethnic OR AB ethnic

S106 TI "cross cultural" OR AB "cross cultural"

S105 TI Caucasian* OR AB Caucasian*

S104 TI Blacks OR AB Blacks

S103 TI black OR AB black

S102 TI Asian* OR AB Asian*

S101 TI "African American*" OR AB "African American*"

S100 (MH "Ethnic Groups")

S99 (MH "Ethnological Research")

S98 (MH "Whites")

S97 (MH "Arabs")

S96 (MH "Native Americans")

S95 MH "Asians"

S94 (MH "Blacks")

S93 S91 OR S92

S92 S12 AND S90

S91 S12 AND S39

S90 S40 OR S41 OR S42 OR S43 OR S44 OR S45 OR S46 OR S47 OR S48 OR S49 OR S50 OR S51 OR S52 OR S53 OR S54 OR S55 OR S56 OR S57 OR S58 OR S59 OR S60 OR S61 OR S62 OR S63 OR S64 OR S65 OR S66 OR S67 OR S68 OR S69 OR S70 OR S71 OR S72 OR S73 OR S74 OR S75 OR S76 OR S77 OR S78 OR S79 OR S80 OR S81 OR S82 OR S83 OR S84 OR S85 OR S86 OR S87 OR S88 OR S89

S89 TI (rheumatic N2 disease*) OR AB (rheumatic N2 disease*)

S88 (MH "Rheumatic Diseases")

S87 TI fatigue OR AB fatigue

S86 (MH "Fatigue")

S85 TI (episodic N2 disabilit*) OR AB (episodic N2 disabilit*)

S84 TI (disease N2 flare*) OR AB (disease N2 flare*)

S83 TI bullying OR AB bullying

S82 (MH "Bullying")

S81 TI (workplace* N2 violence) OR AB (workplace* N2 violence)

S80 (MH "Workplace Violence")

S79 TI (PTSD or PTSS) OR AB (PTSD or PTSS)

S78 TI "posttraumatic stress" OR AB "posttraumatic stress"

S77 TI "post-traumatic stress" OR AB "post-traumatic stress"

S76 (MH "Stress Disorders, Post-Traumatic")

S75 TI depression OR AB depression

S74 (MH "Depression")

S73 TI anxiety OR AB anxiety

S72 (MH "Anxiety")

S71 TI trauma OR AB trauma

S70 (MH "Trauma")

S69 TI (chronic N2 pain) OR AB (chronic N2 pain)

S68 (MH "Chronic Pain")

S67 TI "low back pain" OR AB "low back pain"

S66 (MH "Low Back Pain")

S65 TI cancer OR AB cancer

S64 (MH "Neoplasms")

S63 TI TBI OR AB TBI

S62 TI "traumatic brain injur*" OR AB "traumatic brain injur*"

S61 (MH "Brain Injuries")

S60 TI "spinal cord injur*" OR AB "spinal cord injur*"

S59 (MH "Spinal Cord Injuries")

S58 TI (visual N2 impair*) OR AB (visual N2 impair*)

S57 (MH "Vision Disorders")

S56 TI (hearing N2 impair*) OR AB (hearing N2 impair*)

S55 (MH "Hearing Disorders")

S54 TI (mental N2 illness*) OR AB (mental N2 illness*)

S53 TI amputee# OR AB amputee#

S52 (MH "Amputees")

S51 (MH "Stroke")

S50 TI "cardiovascular disease*" OR AB "cardiovascular disease*"

S49 (MH "Cardiovascular Diseases")

S48 (MH "Cardiovascular Diseases+")

S47 (MH "Mental Disorders")

S46 (MH "Disabled")

S45 (MH "Heart Diseases")

S44 TI "heart disease" OR AB "heart disease"

S43 TI impairment OR AB impairment

S42 TI disabilit* OR AB disabilit*

S41 TI (chronic N2 disease*) OR AB (chronic N2 disease*)

S40 (MH "Chronic Disease")

S39 S13 OR S14 OR S15 OR S16 OR S17 OR S18 OR S19 OR S20 OR S21 OR S22 OR S23 OR S24 OR S25 OR S26 OR S27 OR S28 OR S29 OR S30 OR S31 OR S32 OR S33 OR S34 OR S35 OR S36 OR S37 OR S38

S38 TI "job disruption" OR AB "job disruption"

S37 TI (insurance N2 claim*) OR AB (insurance N2 claim*)

S36 TI "longterm disabilit* OR AB "longterm disabilit*"

S35 TI "long-term disabilit* OR AB "long-term disabilit*"

S34 TI "shortterm disabilit*" OR AB "shortterm disabilit*"

S33 TI "short-term disabilit*" OR AB "short-term disabilit*"

S32 TI claimant* OR AB claimant*

S31 TI illness* OR AB illness*

S30 TI injur* OR AB injur*

S29 TI (acquired N2 condition*) OR AB (acquired N2 condition*)

S28 TI (work* N2 illness*) OR AB (work* N2 illness*)

S27 TI (worker* N2 compensation) OR AB (worker* N2 compensation)

S26 (MH "Worker's Compensation")

S25 TI (work* N2 injur*) OR AB (work* N2 injur*)

S24 TI (work* N2 disabilit*) OR AB (work* N2 disabilit*)

S23 TI presenteeism OR AB presenteeism

S22 (MH "Presenteeism")

S21 TI (non-occupational N2 illness*) OR AB (non-occupational N2 illness*)

S20 TI (occupational N2 illness*) OR AB (occupational N2 illness*)

S19 TI (occupational N2 injur*) OR AB (occupational N2 injur*)

S18 (MH "Occupational-Related Injuries")

S17 TI (occupational N2 rehab*) OR AB (occupational N2 rehab*)

S16 TI (occupational N2 disease*) OR AB (occupational N2 disease*)

S15 (MH "Occupational Diseases")

S14 TI absenteeism AND AB absenteeism

S13 (MH "Absenteeism")

S11 TI "work site*" OR AB "work site*"

S10 TI worksite* OR AB worksite*

S9 MH "Work environment"

S8 MH "Work"

S7 TI occupation* OR AB occupation*

S6 TI job# OR AB job#

S5 TI employment OR AB employment

S4 MH “Employment”

S3 TI employer# OR AB employer#

S2 TI employee# OR AB employee#

S1 TI (Worker# or labo#rer#) OR AB (Worker# or labo#rer#)

*Database 5: ECONLit (EBSCO)*

S214 S63 AND S211 AND S212 Limiters - Published Date: 20010101-20210431

S213 S63 AND S211 AND S212

S212 S106 OR S122

S211 S123 OR S124 OR S125 OR S126 OR S127 OR S128 OR S129 OR S130 OR S131 OR S132 OR S133 OR S134 OR S135 OR S136 OR S137 OR S138 OR S139 OR S140 OR S141 OR S142 OR S143 OR S144 OR S145 OR S146 OR S147 OR S148 OR S149 OR S150 OR S151 OR S152 OR S153 OR S154 OR S155 OR S156 OR S157 OR S158 OR S159 OR S160 OR S161 OR S162 OR S163 OR S164 OR S165 OR S166 OR S167 OR S168 OR S169 OR S170 OR S171 OR S172 OR S173 OR S174 OR S175 OR S176 OR S177 OR S178 OR S179 OR S180 OR S181 OR S182 OR S183 OR S184 OR S185 OR S186 OR S187 OR S188 OR S189 OR S190 OR S191 OR S192 OR S193 OR S194 OR S195 OR S196 OR S197 OR S198 OR S199 OR S200 OR S201 OR S202 OR S203 OR S204 OR S205 OR S206 OR S207 OR S208 OR S209 OR S210

S210 TI ("career path" N2 damag*) OR AB ("career path" N2 damag*)

S209 TI "future earning capacity" OR AB "future earning capacity"

S208 TI (productivity N2 loss*) OR AB (productivity N2 loss*)

S207 TI ("employment opportunit*" N2 loss*) OR AB ("employment opportunit*" N2 loss*)

S206 TI poverty OR AB poverty

S205 TI "financial stress*" OR AB "financial stress*"

S204 TI financial hardship* OR AB financial hardship*

S203 TI (long-term N2 "income loss*") OR AB (long-term N2 "income loss*")

S202 TI (longterm N2 "income loss*") OR AB (longterm N2 "income loss*")

S201 TI (modifi* N2 work) OR AB (modifi* N2 work)

S200 TI (modifi* N2 duties) OR AB (modifi* N2 duties)

S199 TI (modifi* N2 duty) OR AB (modifi* N2 duty)

S198 TI Flexible work* OR AB Flexible work*

S197 TI disability management program* OR AB disability management program

S196 TI alternat* duties OR AB alternat* duties

S195 TI alternat* duty OR AB alternat* duty

S194 TI worklessness OR AB worklessness

S193 TI work readiness OR AB work readiness

S192 TI (work* N2 reintegrat*) OR AB work* N2 reintegrat*)

S191 TI (work* N2 re-integrat*) OR AB work* N2 re-integrat*)

S190 TI (work* N2 participation) OR AB (work* N2 participation)

S189 TI (work* N2 maintenance) OR AB (work* N2 maintenance)

S188 TI (work* N2 limit*) OR AB (work* N2 limit*)

S187 TI (work* N2 disab*) OR AB (work* N2 disab*)

S186 TI work capacity OR AB work capacity

S185 TI workability OR AB workability

S184 TI work ability OR AB work ability

S183 TI (work* N2 accommodat*) OR AB (work* N2 accommodat*)

S182 TI wage replace* OR AB wage replace*

S181 TI (time N1 benefit#) OR AB (time N1 benefit#)

S180 TI time lost OR AB time lost

S179 TI time loss OR AB time loss

S178 TI (support* N2 supervisor#) OR AB (support* N2 supervisor#)

S177 TI (support* N2 manager#) OR AB (support* N2 manager#)

S176 TI (support* N2 colleague#) OR AB (support* N2 colleague#)

S175 TI (support* N2 co-worker#) OR AB (support* N2 co-worker#)

S174 TI (support* N2 coworker#) OR AB (support* N2 coworker#)

S173 TI suitable duty OR AB suitable duty

S172 TI suitable duties OR AB suitable duties

S171 TI social exclusion OR AB social exclusion

S170 TI (sick* N2 absence#) OR AB (sick* N2 absence#)

S169 TI sick list# OR AB sick list#

S168 TI "short-term disabilit*" OR AB "short-term disabilit*"

S167 TI "short term disabilit*" OR AB "short term disabilit*"

S166 TI second* injur* OR AB second* injur*

S165 TI second employer program# OR AB second employer program#

S164 TI (return* N2 work*) OR AB (return* N2 work*)

S163 TI reasonable accommodation# OR AB reasonable accommodation#

S162 TI relapse# OR AB relapse#

S161 TI (redeployed N2 job) OR AB (redeployed N2 job)

S160 TI reinjur* OR AB reinjur*

S159 TI re-injur* OR AB re-injur*

S158 TI re-employ* OR AB re-employ*

S157 TI reemploy* OR AB reemploy*

S156 TI presenteeism OR AB presenteeism

S155 TI "precarious work* arrangement#" OR AB "precarious work* arrangement#"

S154 TI physical capacity OR AB physical capacity

S153 TI (maintenance N2 work) OR AB (maintenance N2 work)

S152 TI (lost N2 workday*) OR AB (lost N2 workday*)

S151 TI (lost N2 "work day*") OR AB (lost N2 "work day*")

S150 TI "lost time" OR AB "lost time"

S149 TI "long-term insurance" OR AB "long-term insurance"

S148 TI "longterm disabilit*" OR AB "longterm disabilit*"

S147 TI "long-term disabilit*" OR AB "long-term disabilit*"

S146 TI liability reduction OR AB liability reduction

S145 TI "labo#r market re-entry" OR AB "labo#r market re-entry"

S144 TI "labo#r market reentry" OR AB "labo#r market reentry"

S143 TI (injur# N2 experience#) OR AB (injur# N2 experience#)

S142 TI health-related work role functioning OR AB health-related work role functioning

S141 (ZU "health: general")

S140 TI graded activit* OR AB graded activit*

S139 TI functional limitation# OR AB functional limitation#

S138 TI fit note# OR AB fit note#

S137 TI continuance rate# OR AB continuance rate#

S136 TI continuance cost# OR AB continuance cost#

S135 TI compensation cost# OR AB compensation cost#

S134 TI (claim* N2 cost#) OR AB (claim* N2 cost#)

S133 TI "compensation claim# cost#" OR AB "compensation claim# cost#"

S132 TI (communicat* N2 workplace#) OR AB (communicat* N2 workplace#)

S131 TI (communicat* N2 "work place#") OR AB (communicat* N2 "work place#")

S130 TI (communicat* N2 healthcare) OR AB (communicat* N2 healthcare)

S129 TI (communicat* N2 "health care") OR AB (communicat* N2 "health care")

S128 TI (communicat* N2 employer#) OR AB (communicat* N2 employer#)

S127 TI (benefit* N2 duration) OR AB (benefit* N2 duration)

S126 TI (attendance N2 work) OR AB (attendance N2 work)

S125 TI "alternative task*" OR AB "alternative task*"

S124 TI "alternative work*" OR AB "alternative work*"

S123 TI absenteeism OR AB absenteeism

S122 S107 OR S108 OR S109 OR S110 OR S111 OR S112 OR S113 OR S114 OR S115 OR S116 OR S117

S121 TI racial OR AB racial

S120 TI immigra* OR AB immigra*

S119 TI refugee* OR AB refugee*

S118 TI migra* OR AB migra*

S117 TI religio* OR AB religio*

S116 TI cultur* OR AB cultur*

S115 TI oppress* OR AB oppress*

S114 TI "unfair treat*" OR AB "unfair treat*"

S113 TI bully* OR AB bully*

S112 TI harass* OR AB harass*

S111 TI hostil* OR AB hostil*

S110 TI prejud* OR AB prejud*

S109 TI bias OR AB bias

S108 TI discrimin* OR AB discrimin*

S107 TI racis* OR AB racis*

S106 S100 OR S105

S105 S101 NOT S104

S104 S102 OR S103

S103 (ZU "india")

S102 ( TI India OR AB India ) OR ( TI India's OR AB India's )

S101 TI Indian# OR AB Indian#

S100 S64 OR S65 OR S66 OR S67 OR S68 OR S69 OR S70 OR S71 OR S72 OR S73 OR S74 OR S75 OR S76 OR S77 OR S78 OR S79 OR S80 OR S81 OR S82 OR S83 OR S84 OR S85 OR S86 OR S87 OR S88 OR S89 OR S90 OR S91 OR S92 OR S93 OR S94 OR S95 OR S96 OR S97 OR S98 OR S99

S99 TI non-white OR AB non-white

S98 TI "visible minorit*" OR AB "visible minorit*"

S97 TI (Saami or Sami) OR AB (Saami or Sami)

S96 TI "North American native#" OR AB "North American native#"

S95 TI (tribal or tribe#) OR AB (tribal or tribe#)

S94 TI "Native population" OR AB "Native population"

S93 TI "Native people#" OR AB "Native people#"

S92 TI "Native Hawaiian*" OR AB "Native Hawaiian*"

S91 TI "Native Canadian*" OR AB "Native Canadian*"

S90 TI Eskimo# OR AB Eskimo#

S89 TI Amerindian# OR AB Amerindian#

S88 TI "American Indian#" OR AB "American Indian#"

S87 TI "First People#" OR AB "First People#"

S86 TI "First Nation#" OR AB "First Nation#"

S85 TI Maori* OR AB Maori*

S84 TI Inuit OR AB Inuit

S83 TI Metis OR AB Metis

S82 TI Aboriginal* OR AB Aboriginal*

S81 TI Indigenous OR AB Indigenous

S80 TI Caribbean OR AB Caribbean

S79 TI whites OR AB whites

S78 TI white OR AB white

S77 TI racial OR AB racial

S76 TI races OR AB races

S75 TI race OR AB race

S74 TI "Native American*" OR AB "Native American*"

S73 TI Latino* OR AB Latino*

S72 TI Latina* OR AB Latina*

S71 TI hispanic* OR AB hispanic*

S70 TI ethnic* OR AB ethnic*

S69 TI "cross cultural" OR AB "cross cultural"

S68 TI Caucasian* OR AB Caucasian*

S67 TI Blacks OR AB Blacks

S66 TI black OR AB black

S65 TI Asian* OR AB Asian*

S64 TI "African American*" OR AB "African American*"

S63 S61 OR S62

S62 S10 AND S60

S61 S10 AND S33

S60 S34 OR S35 OR S36 OR S37 OR S38 OR S39 OR S40 OR S41 OR S42 OR S43 OR S44 OR S45 OR S46 OR S47 OR S48 OR S49 OR S50 OR S51 OR S52 OR S53 OR S54 OR S55 OR S56 OR S57 OR S58 OR S59

S59 TI (rheumatic N2 disease*) OR AB (rheumatic N2 disease*)

S58 TI fatigue OR AB fatigue

S57 TI (episodic N2 disabilit*) OR AB (episodic N2 disabilit*)

S56 TI (disease N2 flare*) OR AB (disease N2 flare*)

S55 TI bullying OR AB bullying

S54 TI (workplace* N2 violence) OR AB (workplace* N2 violence)

S53 TI (PTSD or PTSS) OR AB (PTSD or PTSS)

S52 TI "posttraumatic stress" OR AB "posttraumatic stress"

S51 TI "post-traumatic stress" OR AB "post-traumatic stress"

S50 TI depression OR AB depression

S49 TI anxiety OR AB anxiety

S48 TI trauma OR AB trauma

S47 TI (chronic N2 pain) OR AB (chronic N2 pain)

S46 TI "low back pain" OR AB "low back pain"

S45 TI cancer OR AB cancer

S44 TI TBI OR AB TBI

S43 TI (visual N2 impair*) OR AB (visual N2 impair*)

S42 TI (hearing N2 impair*) OR AB (hearing N2 impair*)

S41 TI (mental N2 illness*) OR AB (mental N2 illness*)

S40 TI amputee# OR AB amputee#

S39 TI stroke OR AB stroke

S38 TI "cardiovascular disease*" OR AB "cardiovascular disease*"

S37 TI "heart disease" OR AB "heart disease"

S36 TI impairment OR AB impairment

S35 TI disabilit* OR AB disabilit*

S34 TI (chronic N2 disease*) OR AB (chronic N2 disease*)

S33 S11 OR S12 OR S13 OR S14 OR S15 OR S16 OR S17 OR S18 OR S19 OR S20 OR S21 OR S22 OR S23 OR S24 OR S25 OR S26 OR S27 OR S28 OR S29 OR S30 OR S31 OR S32

S32 TI "job disruption" OR AB "job disruption"

S31 TI (insurance N2 claim*) OR AB (insurance N2 claim*)

S30 TI "longterm disabilit*" OR AB "longterm disabilit*"

S29 TI "long-term disabilit*" OR AB "long-term disabilit*"

S28 TI "shortterm disabilit*" OR AB "shortterm disabilit*"

S27 TI "short-term disabilit*" OR AB "short-term disabilit*"

S26 TI claimant* OR AB claimant*

S25 TI illness* OR AB illness*

S24 TI injur* OR AB injur*

S23 TI (acquired N2 condition*) OR AB (acquired N2 condition*)

S22 TI (work* N2 illness*) OR AB (work* N2 illness*)

S21 TI (worker* N2 compensation) OR AB (worker* N2 compensation)

S20 (ZU "workmen's compensation")

S19 TI (work* N2 injur*) OR AB (work* N2 injur*)

S18 TI (work* N2 disabilit*) OR AB (work* N2 disabilit*)

S17 TI presenteeism OR AB presenteeism

S16 TI (non-occupational N2 illness*) OR AB (non-occupational N2 illness*)

S15 TI (occupational N2 illness*) OR AB (occupational N2 illness*)

S14 TI (occupational N2 injur*) OR AB (occupational N2 injur*)

S13 TI (occupational N2 rehab*) OR AB (occupational N2 rehab*)

S12 TI (occupational N2 disease*) OR AB (occupational N2 disease*) 61

S11 TI absenteeism AND AB absenteeism

S10 S1 OR S2 OR S3 OR S4 OR S5 OR S6 OR S7 OR S8 OR S9

S9 TI "work site*" OR AB "work site*"

S8 TI worksite* OR AB worksite*

S7 TI occupation* OR AB occupation*

S6 (ZU "employment. unemployment.")

S5 TI job# OR AB job#

S4 TI employment OR AB employment

S3 TI employer# OR AB employer#

S2 TI employee# OR AB employee#

S1 ( TI (Worker# or labo#rer#) ) OR ( AB (Worker# or labo#rer#) )

*Database 6: ABI Inform (ProQuest)*

Note: Due to the limitations of ProQuest in combining large strings of terms, this search was run twice with the outcomes divided into two sets, and the results were then combined.

Search 1:

S1

ti,ab(worker*) OR ti,ab(employee*) OR ti,ab(employer*) OR ti,ab(employment) OR ti,ab(job*) OR ti,ab(occupation*) OR MAINSUBJECT.EXACT("Employment") OR MAINSUBJECT.EXACT("Work") OR MAINSUBJECT.EXACT("Workplaces") OR ti,ab(worksite*) OR ti,ab("work site*")

S2

MAINSUBJECT.EXACT("Absenteeism") OR ti,ab(absenteeism) OR MAINSUBJECT.EXACT("Occupational diseases") OR ti,ab(presenteeism) OR MAINSUBJECT.EXACT("Workers compensation") OR ti,ab(worker* NEAR/2 compensation) OR ti,ab(acquired NEAR/2 condition*) OR MAINSUBJECT.EXACT("Occupational accidents") OR ti,ab(injur* OR illness* OR claimant*) OR ti,ab(insurance NEAR/2 claim*) OR ti,ab(“job disruption")

S3

MAINSUBJECT.EXACT("Chronic illnesses") OR ti,ab(chronic NEAR/2 disease*) OR ti,ab(impairment) OR ti,ab(“heart disease”) OR MAINSUBJECT.EXACT("Cardiovascular disease") OR MAINSUBJECT.EXACT("Mental disorders") OR ti,ab(("cardiovascular disease" OR "cardiovascular diseases")) OR MAINSUBJECT.EXACT("Stroke") OR ti,ab(stroke) OR MAINSUBJECT.EXACT("Traumatic brain injury") OR ti,ab(TBI) OR MAINSUBJECT.EXACT("Cancer") OR ti,ab(cancer) OR MAINSUBJECT.EXACT("Back pain") OR ti,ab(“low back pain”) OR ti,ab(chronic NEAR/2 pain) OR MAINSUBJECT.EXACT("Trauma") OR ti,ab(trauma) OR MAINSUBJECT.EXACT("Anxieties") OR ti,ab(anxiety) OR MAINSUBJECT.EXACT("Mental depression") OR ti,ab(depression) OR MAINSUBJECT.EXACT("Post traumatic stress disorder") OR ti,ab(“post-traumatic stress”) OR ti,ab(“posttraumatic stress”) OR ti,ab(PTSD or PTSS) OR MAINSUBJECT.EXACT("Workplace violence") OR ti,ab(workplace* NEAR/2 violence) OR MAINSUBJECT.EXACT("Bullying") OR ti,ab(bullying) OR ti,ab(disease NEAR/2 flare*) OR MAINSUBJECT.EXACT("Fatigue") OR ti,ab(fatigue) OR MAINSUBJECT.EXACT("Rheumatic diseases") OR ti,ab(rheumatic NEAR/2 disease*)

S4

S1 AND S2

S5

S1 AND S3

S6

S4 OR S5

S7

MAINSUBJECT.EXACT("African Americans") OR MAINSUBJECT.EXACT("Blacks") OR MAINSUBJECT.EXACT("Asians") OR MAINSUBJECT.EXACT("Arabs") MAINSUBJECT.EXACT("Minority & ethnic groups") OR MAINSUBJECT.EXACT("Whites") OR ti,ab(("african american" OR "african americans")) OR ti,ab(Asian*) OR ti,ab(Black or Blacks) OR ti,ab(Caucasian*) OR ti,ab(cross cultural) OR ti,ab(ethnic or ethnicity) OR ti,ab(Hispanic*) OR ti,ab(Latina* OR Latino*) OR ti,ab(("native american" OR "native americans")) OR ti,ab(race OR races) OR ti,ab(white or whites) OR ti,ab(Caribbean) OR ti,ab(Aboriginal*) OR ti,ab(Metis) OR ti,ab(Inuit) OR ti,ab(Maori*) OR ti,ab(("first nation" OR "first national" OR "first nationalist" OR "first nationally" OR "first nationals" OR "first nations" OR "first nationwide")) OR ti,ab(("first people" OR "first peoples")) OR ti,ab(("american indian" OR "american indians")) OR ti,ab(Amerindian*) OR ti,ab(Eskimo*) OR ti,ab(("native canadian" OR "native canadians")) OR ti,ab(("native hawaiian" OR "native hawaiians")) OR ti,ab(("native people" OR "native peoples")) OR ti,ab(("native population" OR "native populations")) OR ti,ab(tribal or tribe) OR ti,ab(“North American Native”) OR ti,ab(Saami OR Sami) OR ti,ab(("visible minorities" OR "visible minority")) OR ti,ab(non-white) OR (ti,ab(Indian*) NOT (ti,ab(India OR India's)))

S8

MAINSUBJECT.EXACT("Racism") OR ti,ab(racis*) OR ti,ab(discrim*) OR ti,ab(bias) OR ti,ab(prejud*) OR ti,ab(hostil*) OR ti,ab(harass*) OR ti,ab(("unfair treatment")) OR ti,ab(oppress*) OR MAINSUBJECT.EXACT("Prejudice") OR ti,ab(cultur*) OR ti,ab(religio*) OR ti,ab(migra*) OR ti,ab(refugee*) OR MAINSUBJECT.EXACT("Minority Groups") OR ti,ab(immigra*) OR MAINSUBJECT.EXACT("Immigrants") OR MAINSUBJECT.EXACT("Undocumented Immigrants") OR ti,ab(racial) OR ti,ab(segregation)

S9

S7 OR S8

S10

ti,ab("alternative work*" OR "alternative task*") OR ti,ab(attendance NEAR/2 work) OR ti,ab(benefit* NEAR/2 duration) OR ti,ab(communicat* NEAR/2 (employer* OR "health care" OR healthcare OR "work place*" OR workplace*)) OR ti,ab("compensation claim* cost*") OR ti,ab(claim* NEAR/2 cost*) OR ti,ab("compensation cost" OR "continuance cost" OR "continuance rate") OR ti,ab("fit note*" OR "functional limitation") OR MAINSUBJECT.EXACT("Health") OR ti,ab("labo?r market reentry" OR "labo?r market re-entry") OR ti,ab("liability reduction" OR "lost time") OR ti,ab(lost NEAR/2 "work day*") OR ti,ab(maintenance NEAR/2 work) OR ti,ab("physical capacity" OR "precarious work* arrangement*" OR presenteeism) OR ti,ab(re-employ* OR reemploy* OR re-injur* OR reinjur*) OR ti,ab("reasonable accommodation" OR relapse*) OR ti,ab(return NEAR/3 work*)

S11

S6 AND S9 AND S10

S12

(S6 AND S9 AND S10) AND PEER(yes)

S13

(S6 AND S9 AND S10) AND (pd(20010101-20210430) AND PEER(yes))

Search 2:

S1

ti,ab(worker*) OR ti,ab(employee*) OR ti,ab(employer*) OR ti,ab(employment) OR ti,ab(job*) OR ti,ab(occupation*) OR MAINSUBJECT.EXACT("Employment") OR MAINSUBJECT.EXACT("Work") OR MAINSUBJECT.EXACT("Workplaces") OR ti,ab(worksite*) OR ti,ab("work site*")

S2

MAINSUBJECT.EXACT("Absenteeism") OR ti,ab(absenteeism) OR MAINSUBJECT.EXACT("Occupational diseases") OR ti,ab(presenteeism) OR MAINSUBJECT.EXACT("Workers compensation") OR ti,ab(worker* NEAR/2 compensation) OR ti,ab(acquired NEAR/2 condition*) OR MAINSUBJECT.EXACT("Occupational accidents") OR ti,ab(injur* OR illness* OR claimant*) OR ti,ab(insurance NEAR/2 claim*) OR ti,ab(“job disruption")

S3

MAINSUBJECT.EXACT("Chronic illnesses") OR ti,ab(chronic NEAR/2 disease*) OR ti,ab(impairment) OR ti,ab(“heart disease”) OR MAINSUBJECT.EXACT("Cardiovascular disease") OR MAINSUBJECT.EXACT("Mental disorders") OR ti,ab(("cardiovascular disease" OR "cardiovascular diseases")) OR MAINSUBJECT.EXACT("Stroke") OR ti,ab(stroke) OR MAINSUBJECT.EXACT("Traumatic brain injury") OR ti,ab(TBI) OR MAINSUBJECT.EXACT("Cancer") OR ti,ab(cancer) OR MAINSUBJECT.EXACT("Back pain") OR ti,ab(“low back pain”) OR ti,ab(chronic NEAR/2 pain) OR MAINSUBJECT.EXACT("Trauma") OR ti,ab(trauma) OR MAINSUBJECT.EXACT("Anxieties") OR ti,ab(anxiety) OR MAINSUBJECT.EXACT("Mental depression") OR ti,ab(depression) OR MAINSUBJECT.EXACT("Post traumatic stress disorder") OR ti,ab(“post-traumatic stress”) OR ti,ab(“posttraumatic stress”) OR ti,ab(PTSD or PTSS) OR MAINSUBJECT.EXACT("Workplace violence") OR ti,ab(workplace* NEAR/2 violence) OR MAINSUBJECT.EXACT("Bullying") OR ti,ab(bullying) OR ti,ab(disease NEAR/2 flare*) OR MAINSUBJECT.EXACT("Fatigue") OR ti,ab(fatigue) OR MAINSUBJECT.EXACT("Rheumatic diseases") OR ti,ab(rheumatic NEAR/2 disease*)

S4

S1 AND S2

S5

S1 AND S3

S6

S4 OR S5

S7

MAINSUBJECT.EXACT("African Americans") OR MAINSUBJECT.EXACT("Blacks") OR MAINSUBJECT.EXACT("Asians") OR MAINSUBJECT.EXACT("Arabs") MAINSUBJECT.EXACT("Minority & ethnic groups") OR MAINSUBJECT.EXACT("Whites") OR ti,ab(("african american" OR "african americans")) OR ti,ab(Asian*) OR ti,ab(Black or Blacks) OR ti,ab(Caucasian*) OR ti,ab(cross cultural) OR ti,ab(ethnic or ethnicity) OR ti,ab(Hispanic*) OR ti,ab(Latina* OR Latino*) OR ti,ab(("native american" OR "native americans")) OR ti,ab(race OR races) OR ti,ab(white or whites) OR ti,ab(Caribbean) OR ti,ab(Aboriginal*) OR ti,ab(Metis) OR ti,ab(Inuit) OR ti,ab(Maori*) OR ti,ab(("first nation" OR "first national" OR "first nationalist" OR "first nationally" OR "first nationals" OR "first nations" OR "first nationwide")) OR ti,ab(("first people" OR "first peoples")) OR ti,ab(("american indian" OR "american indians")) OR ti,ab(Amerindian*) OR ti,ab(Eskimo*) OR ti,ab(("native canadian" OR "native canadians")) OR ti,ab(("native hawaiian" OR "native hawaiians")) OR ti,ab(("native people" OR "native peoples")) OR ti,ab(("native population" OR "native populations")) OR ti,ab(tribal or tribe) OR ti,ab(“North American Native”) OR ti,ab(Saami OR Sami) OR ti,ab(("visible minorities" OR "visible minority")) OR ti,ab(non-white) OR (ti,ab(Indian*) NOT (ti,ab(India OR India's)))

S8

MAINSUBJECT.EXACT("Racism") OR ti,ab(racis*) OR ti,ab(discrim*) OR ti,ab(bias) OR ti,ab(prejud*) OR ti,ab(hostil*) OR ti,ab(harass*) OR ti,ab(("unfair treatment")) OR ti,ab(oppress*) OR MAINSUBJECT.EXACT("Prejudice") OR ti,ab(cultur*) OR ti,ab(religio*) OR ti,ab(migra*) OR ti,ab(refugee*) OR MAINSUBJECT.EXACT("Minority Groups") OR ti,ab(immigra*) OR MAINSUBJECT.EXACT("Immigrants") OR MAINSUBJECT.EXACT("Undocumented Immigrants") OR ti,ab(racial) OR ti,ab(segregation)

S9

S7 OR S8

S10

MAINSUBJECT.EXACT("Sick leave") OR ti,ab("sick list*" OR "sick leave") OR ti,ab(sick* NEAR/2 absence*) OR ti,ab("social exclusion" OR "suitable duty" OR "suitable duties" OR "suitable employment") OR ti,ab(support NEAR/2 (coworker* OR co-worker* OR colleague* OR manager* OR supervisor*)) OR ti,ab("time loss" OR "time lost") OR ti,ab(time NEAR/1 benefit*) OR ti,ab("wage replace*") OR ti,ab(work* NEAR/2 (accommodat* OR limit* OR maintenance OR participation OR re-integrat* OR reintegrat*)) OR ti,ab("work capacity" OR "work readiness" OR worklessness) OR ti,ab("disability management program*") OR MAINSUBJECT.EXACT("Case Management") OR ti,ab("flexible work*") OR ti,ab(modifi* NEAR/2 (duty OR duties OR work)) OR ti,ab(“financial hardship*”) OR ti,ab(“financial stress*”) OR ti,ab(poverty) OR MAINSUBJECT.EXACT("Poverty") OR ti,ab(“employment opportunit*" NEAR/2 loss*) OR ti,ab(“future earning capacity”)

S11

S6 AND S9 AND S10

S12

(S6 AND S9 AND S10) AND PEER(yes)

S13

(S6 AND S9 AND S10) AND (pd(20010101-20210430) AND PEER(yes))

*Database 7: ASSIA*

Note: Due to the limitations of ProQuest in combining large strings of terms, this search was run twice with the outcomes divided into two sets, and the results were then combined.

Search 1:

S1

ti,ab(worker*) OR ti,ab(employee*) OR ti,ab(employer*) OR ti,ab(employment) OR ti,ab(job*) OR ti,ab(occupation*) OR MAINSUBJECT.EXACT("Employment") OR MAINSUBJECT.EXACT("Work") OR MAINSUBJECT.EXACT("Workplaces") OR ti,ab(worksite*) OR ti,ab("work site*")

S2

MAINSUBJECT.EXACT("Absenteeism") OR ti,ab(absenteeism) OR MAINSUBJECT.EXACT("Occupational diseases") OR ti,ab(presenteeism) OR MAINSUBJECT.EXACT("Workers compensation") OR ti,ab(worker* NEAR/2 compensation) OR ti,ab(acquired NEAR/2 condition*) OR MAINSUBJECT.EXACT("Industrial accidents") OR ti,ab(injur* OR illness* OR claimant*) OR ti,ab(insurance NEAR/2 claim*) OR ti,ab(“job disruption")

S3

MAINSUBJECT.EXACT("Chronic diseases") OR ti,ab(chronic NEAR/2 disease*) OR ti,ab(impairment) OR ti,ab(“heart disease”) OR MAINSUBJECT.EXACT("Heart diseases") OR MAINSUBJECT.EXACT("Psychiatric disorders") OR MAINSUBJECT.EXACT("Cardiovascular diseases") OR ti,ab(("cardiovascular disease" OR "cardiovascular diseases")) OR MAINSUBJECT.EXACT("Strokes") OR ti,ab(stroke) OR MAINSUBJECT.EXACT("Traumatic brain injury") OR ti,ab(TBI) OR MAINSUBJECT.EXACT("Cancer") OR ti,ab(cancer) OR MAINSUBJECT.EXACT("Low back pain") OR ti,ab(“low back pain”) OR ti,ab(chronic NEAR/2 pain) OR MAINSUBJECT.EXACT("Psychological trauma") OR ti,ab(trauma) OR MAINSUBJECT.EXACT("Anxiety") OR ti,ab(anxiety) OR MAINSUBJECT.EXACT("Depression") OR ti,ab(depression) OR MAINSUBJECT.EXACT("Posttraumatic stress disorder") OR ti,ab(“post-traumatic stress”) OR ti,ab(“posttraumatic stress”) OR ti,ab(PTSD or PTSS) OR MAINSUBJECT.EXACT("Workplace violence") OR ti,ab(workplace* NEAR/2 violence) OR MAINSUBJECT.EXACT("Bullying") OR ti,ab(bullying) OR ti,ab(disease NEAR/2 flare*) OR MAINSUBJECT.EXACT("Fatigue") OR ti,ab(fatigue) OR MAINSUBJECT.EXACT("Rheumatic diseases") OR ti,ab(rheumatic NEAR/2 disease*)

S4

S1 AND S2

S5

S1 AND S3

S6

S4 OR S5

S7

MAINSUBJECT.EXACT("Black American people") OR MAINSUBJECT.EXACT("Black people") OR MAINSUBJECT.EXACT("Asian people") OR MAINSUBJECT.EXACT("Arabs") OR MAINSUBJECT.EXACT("Ethnic groups") OR MAINSUBJECT.EXACT("White people") OR ti,ab(“African American*”) OR ti,ab(Asian*) OR ti,ab(Black or Blacks) OR ti,ab(Caucasian*) OR ti,ab(cross cultural) OR ti,ab(ethnic or ethnicity) OR ti,ab(Hispanic*) OR ti,ab(Latina* OR Latino*) OR ti,ab(“Native American*”) OR ti,ab(race OR races) OR ti,ab(white or whites) OR ti,ab(Caribbean) OR ti,ab(Aboriginal*) OR ti,ab(Metis) OR ti,ab(Inuit) OR ti,ab(Maori*) OR ti,ab(“First Nation*”) OR ti,ab(“First People*”) OR ti,ab(“American Indian*”) OR ti,ab(Amerindian*) OR ti,ab(Eskimo*) OR ti,ab(“Native Canadian*”) OR ti,ab(“Native Hawaiian*”) OR ti,ab(“Native people*”) OR ti,ab(“Native population*”) OR ti,ab(tribal or tribe) OR ti,ab(“North American Native”) OR ti,ab(Saami OR Sami) OR ti,ab(“visible minorit*”) OR ti,ab(non-white) OR (ti,ab(Indian*) NOT (ti,ab(India OR India's) OR MAINSUBJECT.EXACT("India")))

S8

MAINSUBJECT.EXACT("Racism") OR ti,ab(racis*) OR ti,ab(discrim*) OR ti,ab(bias) OR ti,ab(prejud*) OR ti,ab(hostil*) OR ti,ab(harass*) OR ti,ab(("unfair treatment")) OR ti,ab(oppress*) OR MAINSUBJECT.EXACT("Prejudice") OR ti,ab(cultur*) OR ti,ab(religio*) OR ti,ab(migra*) OR ti,ab(refugee*) OR MAINSUBJECT.EXACT("Minority Groups") OR ti,ab(immigra*) OR MAINSUBJECT.EXACT("Immigrants") OR MAINSUBJECT.EXACT("Illegal Immigrants") OR ti,ab(racial) OR ti,ab(segregation)

S9

S7 OR S8

S10

ti,ab("alternative work*" OR "alternative task*") OR ti,ab(attendance NEAR/2 work) OR ti,ab(benefit* NEAR/2 duration) OR ti,ab(communicat* NEAR/2 (employer* OR "health care" OR healthcare OR "work place*" OR workplace*)) OR ti,ab("compensation claim* cost*") OR ti,ab(claim* NEAR/2 cost*) OR ti,ab("compensation cost" OR "continuance cost" OR "continuance rate") OR ti,ab("fit note*" OR "functional limitation") OR MAINSUBJECT.EXACT("Health") OR ti,ab("labo?r market reentry" OR "labo?r market re-entry") OR ti,ab("liability reduction" OR "lost time") OR ti,ab(lost NEAR/2 "work day*") OR ti,ab(maintenance NEAR/2 work) OR ti,ab("physical capacity" OR "precarious work* arrangement*" OR presenteeism) OR ti,ab(re-employ* OR reemploy* OR re-injur* OR reinjur*) OR ti,ab("reasonable accommodation" OR relapse*) OR ti,ab(return NEAR/3 work*)

S11

S6 AND S9 AND S10

S12

(S6 AND S9 AND S10) AND PEER(yes)

S13

(S6 AND S9 AND S10) AND (pd(20010101-20210430) AND PEER(yes))

Search 2:

S1

ti,ab(worker*) OR ti,ab(employee*) OR ti,ab(employer*) OR ti,ab(employment) OR ti,ab(job*) OR ti,ab(occupation*) OR MAINSUBJECT.EXACT("Employment") OR MAINSUBJECT.EXACT("Work") OR MAINSUBJECT.EXACT("Workplaces") OR ti,ab(worksite*) OR ti,ab("work site*")

S2

MAINSUBJECT.EXACT("Absenteeism") OR ti,ab(absenteeism) OR MAINSUBJECT.EXACT("Occupational diseases") OR ti,ab(presenteeism) OR MAINSUBJECT.EXACT("Workers compensation") OR ti,ab(worker* NEAR/2 compensation) OR ti,ab(acquired NEAR/2 condition*) OR MAINSUBJECT.EXACT("Industrial accidents") OR ti,ab(injur* OR illness* OR claimant*) OR ti,ab(insurance NEAR/2 claim*) OR ti,ab(“job disruption")

S3

MAINSUBJECT.EXACT("Chronic diseases") OR ti,ab(chronic NEAR/2 disease*) OR ti,ab(impairment) OR ti,ab(“heart disease”) OR MAINSUBJECT.EXACT("Heart diseases") OR MAINSUBJECT.EXACT("Psychiatric disorders") OR MAINSUBJECT.EXACT("Cardiovascular diseases") OR ti,ab(("cardiovascular disease" OR "cardiovascular diseases")) OR MAINSUBJECT.EXACT("Strokes") OR ti,ab(stroke) OR MAINSUBJECT.EXACT("Traumatic brain injury") OR ti,ab(TBI) OR MAINSUBJECT.EXACT("Cancer") OR ti,ab(cancer) OR MAINSUBJECT.EXACT("Low back pain") OR ti,ab(“low back pain”) OR ti,ab(chronic NEAR/2 pain) OR MAINSUBJECT.EXACT("Psychological trauma") OR ti,ab(trauma) OR MAINSUBJECT.EXACT("Anxiety") OR ti,ab(anxiety) OR MAINSUBJECT.EXACT("Depression") OR ti,ab(depression) OR MAINSUBJECT.EXACT("Posttraumatic stress disorder") OR ti,ab(“post-traumatic stress”) OR ti,ab(“posttraumatic stress”) OR ti,ab(PTSD or PTSS) OR MAINSUBJECT.EXACT("Workplace violence") OR ti,ab(workplace* NEAR/2 violence) OR MAINSUBJECT.EXACT("Bullying") OR ti,ab(bullying) OR ti,ab(disease NEAR/2 flare*) OR MAINSUBJECT.EXACT("Fatigue") OR ti,ab(fatigue) OR MAINSUBJECT.EXACT("Rheumatic diseases") OR ti,ab(rheumatic NEAR/2 disease*)

S4

S1 AND S2

S5

S1 AND S3

S6

S4 OR S5

S7

MAINSUBJECT.EXACT("Black American people") OR MAINSUBJECT.EXACT("Black people") OR MAINSUBJECT.EXACT("Asian people") OR MAINSUBJECT.EXACT("Arabs") OR MAINSUBJECT.EXACT("Ethnic groups") OR MAINSUBJECT.EXACT("White people") OR ti,ab(“African American*”) OR ti,ab(Asian*) OR ti,ab(Black or Blacks) OR ti,ab(Caucasian*) OR ti,ab(cross cultural) OR ti,ab(ethnic or ethnicity) OR ti,ab(Hispanic*) OR ti,ab(Latina* OR Latino*) OR ti,ab(“Native American*”) OR ti,ab(race OR races) OR ti,ab(white or whites) OR ti,ab(Caribbean) OR ti,ab(Aboriginal*) OR ti,ab(Metis) OR ti,ab(Inuit) OR ti,ab(Maori*) OR ti,ab(“First Nation*”) OR ti,ab(“First People*”) OR ti,ab(“American Indian*”) OR ti,ab(Amerindian*) OR ti,ab(Eskimo*) OR ti,ab(“Native Canadian*”) OR ti,ab(“Native Hawaiian*”) OR ti,ab(“Native people*”) OR ti,ab(“Native population*”) OR ti,ab(tribal or tribe) OR ti,ab(“North American Native”) OR ti,ab(Saami OR Sami) OR ti,ab(“visible minorit*”) OR ti,ab(non-white) OR (ti,ab(Indian*) NOT (ti,ab(India OR India's) OR MAINSUBJECT.EXACT("India")))

S8

MAINSUBJECT.EXACT("Racism") OR ti,ab(racis*) OR ti,ab(discrim*) OR ti,ab(bias) OR ti,ab(prejud*) OR ti,ab(hostil*) OR ti,ab(harass*) OR ti,ab(("unfair treatment")) OR ti,ab(oppress*) OR MAINSUBJECT.EXACT("Prejudice") OR ti,ab(cultur*) OR ti,ab(religio*) OR ti,ab(migra*) OR ti,ab(refugee*) OR MAINSUBJECT.EXACT("Minority Groups") OR ti,ab(immigra*) OR MAINSUBJECT.EXACT("Immigrants") OR MAINSUBJECT.EXACT("Illegal Immigrants") OR ti,ab(racial) OR ti,ab(segregation)

S9

S7 OR S8

S10

MAINSUBJECT.EXACT("Sick leave") OR ti,ab("sick list*" OR "sick leave") OR ti,ab(sick* NEAR/2 absence*) OR ti,ab("social exclusion" OR "suitable duty" OR "suitable duties" OR "suitable employment") OR ti,ab(support NEAR/2 (coworker* OR co-worker* OR colleague* OR manager* OR supervisor*)) OR ti,ab("time loss" OR "time lost") OR ti,ab(time NEAR/1 benefit*) OR ti,ab("wage replace*") OR ti,ab(work* NEAR/2 (accommodat* OR limit* OR maintenance OR participation OR re-integrat* OR reintegrat*)) OR ti,ab("work capacity" OR "work readiness" OR worklessness) OR ti,ab("disability management program*") OR MAINSUBJECT.EXACT("Case Management") OR ti,ab("flexible work*") OR ti,ab(modifi* NEAR/2 (duty OR duties OR work)) OR ti,ab(“financial hardship*”) OR ti,ab(“financial stress*”) OR ti,ab(poverty) OR MAINSUBJECT.EXACT("Poverty") OR ti,ab(“employment opportunit*" NEAR/2 loss*) OR ti,ab(“future earning capacity”)

S11

S6 AND S9 AND S10

S12

(S6 AND S9 AND S10) AND PEER(yes)

S13

(S6 AND S9 AND S10) AND (pd(20010101-20210430) AND PEER(yes))

*Database 8: Sociological Abstracts*

Note: Due to the limitations of ProQuest in combining large strings of terms, this search was run twice with the outcomes divided into two sets, and the results were then combined.

Search 1:

S1

(ti,ab(worker*) OR ti,ab(employee*) OR ti,ab(employer*) OR ti,ab(employment) OR ti,ab(job*) OR ti,ab(occupation*) OR MAINSUBJECT.EXACT("Employment") OR MAINSUBJECT.EXACT("Work") OR

MAINSUBJECT.EXACT("Workplaces") OR ti,ab(worksite*) OR ti,ab("work site*")) AND PEER(yes)

S2

MAINSUBJECT.EXACT("Absenteeism") OR ti,ab(absenteeism) OR MAINSUBJECT.EXACT("Occupational diseases") OR ti,ab(presenteeism) OR MAINSUBJECT.EXACT("Workers compensation") OR ti,ab(worker* NEAR/2 compensation) OR ti,ab(acquired NEAR/2 condition*) OR MAINSUBJECT.EXACT("Occupational accidents") OR ti,ab(injur* OR illness* OR claimant*) OR ti,ab(insurance NEAR/2 claim*) OR ti,ab(“job disruption")

S3

(MAINSUBJECT.EXACT("Chronic Illness") OR ti,ab(chronic NEAR/2 disease*) OR ti,ab(impairment) OR

ti,ab("heart disease") OR MAINSUBJECT.EXACT("Heart Diseases") OR MAINSUBJECT.EXACT("Mental Illness") OR ti,ab("cardiovascular disease*") OR ti,ab(stroke) OR ti,ab(TBI) OR MAINSUBJECT.EXACT("Cancer") OR ti,ab(cancer) OR ti,ab("low back pain") OR ti,ab(chronic NEAR/2 pain) OR MAINSUBJECT.EXACT("Trauma") OR ti,ab(trauma) OR MAINSUBJECT.EXACT("Anxiety") OR ti,ab(anxiety) OR MAINSUBJECT.EXACT("Depression (Psychology)") OR ti,ab(depression) OR MAINSUBJECT.EXACT("Posttraumatic Stress Disorder") OR ti,ab("post-traumatic stress") OR ti,ab("posttraumatic stress") OR ti,ab(PTSD OR PTSS) OR ti,ab(workplace* NEAR/2 violence) OR MAINSUBJECT.EXACT("Aggression") OR ti,ab(bullying) OR ti,ab(disease NEAR/2 flare*) OR MAINSUBJECT.EXACT("Fatigue") OR ti,ab(fatigue) OR MAINSUBJECT.EXACT("Arthrit

is") OR ti,ab(rheumatic NEAR/2 disease*)) AND PEER(yes)

S4

S1 AND S2

S5

S1 AND S3

S6

S4 OR S5

S7

(MAINSUBJECT.EXACT("Black Americans") OR MAINSUBJECT.EXACT("African Cultural Groups") OR MAINSUBJECT.EXACT("Asian Cultural Groups") OR MAINSUBJECT.EXACT("Ethnic Groups") OR MAINSUBJECT.EXACT("Oceanic Cultural Groups") OR MAINSUBJECT.EXACT("European Cultural Groups") OR ti,ab("African American*") OR ti,ab(Asian*) OR ti,ab(Black OR Blacks) OR ti,ab(Caucasian*) OR ti,ab(cross cultural) OR ti,ab(ethnic OR ethnicity) OR ti,ab(Hispanic*) OR ti,ab(Latina* OR Latino*) OR ti,ab("Native American*") OR ti,ab(race OR races) OR ti,ab(white OR whites) OR ti,ab(Caribbean) OR

ti,ab(Aboriginal*) OR ti,ab(Metis) OR ti,ab(Inuit) OR ti,ab(Maori*) OR ti,ab("First Nation*") OR ti,ab("First People*") OR ti,ab("American Indian*") OR ti,ab(Amerindian*) OR ti,ab(Eskimo*) OR ti,ab("Native Canadian*") OR ti,ab("Native Hawaiian*") OR ti,ab("Native people*") OR ti,ab("Native population*") OR ti,ab(tribal OR tribe) OR ti,ab("North American Native") OR ti,ab(Saami OR Sami) OR ti,ab("visible minorit*") OR ti,ab(non-white) OR (ti,ab(Indian*) NOT (ti,ab(India OR India's)))) AND PEER(yes)

S8

(MAINSUBJECT.EXACT("Racism") OR ti,ab(racis*) OR ti,ab(discrim*) OR ti,ab(bias) OR ti,ab(prejud*) OR ti,ab(hostil*) OR ti,ab(harass*) OR ti,ab("unfair treat*") OR ti,ab(oppress*) OR MAINSUBJECT.EXACT("Prejudice") OR ti,ab(cultur*) OR ti,ab(religio*) OR ti,ab(migra*) OR ti,ab(refugee*) MAINSUBJECT.EXACT("Minority Groups") OR ti,ab(immigra*) OR MAINSUBJECT.EXACT("Immigrants") OR MAINSUBJECT.EXACT("Undocumented Immigrants") OR ti,ab(racial) OR ti,ab(segregation)) AND PEER(yes)

S9

S7 OR S8

S10

(ti,ab("alternative work*" OR "alternative task*") OR ti,ab(attendance NEAR/2 work) OR ti,ab(benefit* NEAR/2 duration) OR NEAR/2 duration) OR ti,ab(communicat* NEAR/2 (employer* OR "health care" OR healthcare OR "work place*" OR workplace*)) OR ti,ab("compensation claim* cost*") OR ti,ab(claim*

NEAR/2 cost*) OR ti,ab("compensation cost" OR "continuance cost" OR "continuance rate") OR ti,ab("fit note*" OR "functional limitation") OR MAINSUBJECT.EXACT("Health ") OR ti,ab("labo?r market reentry" OR "labo?r market reentry") OR ti,ab("liability reduction" OR "lost time") OR ti,ab(lost NEAR/2 "work day*") OR ti,ab(maintenance NEAR/2 work) OR ti,ab("physical capacity" OR "precarious work* arrangement*" OR presenteeism) OR ti,ab(reemploy* OR reemploy* OR reinjur* OR reinjur*) OR ti,ab("reasonable accommodation" OR relapse*) OR ti,ab(return NEAR/3 work*)) AND PEER(yes)

S11

S6 AND S9 AND S10

S12

(S6 AND S9 AND S10) AND pd(20010101-20210430)

Search 2:

S1

(ti,ab(worker*) OR ti,ab(employee*) OR ti,ab(employer*) OR ti,ab(employment) OR ti,ab(job*) OR ti,ab(occupation*) OR MAINSUBJECT.EXACT("Employment") OR MAINSUBJECT.EXACT("Work") OR

MAINSUBJECT.EXACT("Workplaces") OR ti,ab(worksite*) OR ti,ab("work site*")) AND PEER(yes)

S2

MAINSUBJECT.EXACT("Absenteeism") OR ti,ab(absenteeism) OR MAINSUBJECT.EXACT("Occupational diseases") OR ti,ab(presenteeism) OR MAINSUBJECT.EXACT("Workers compensation") OR ti,ab(worker* NEAR/2 compensation) OR ti,ab(acquired NEAR/2 condition*) OR MAINSUBJECT.EXACT("Occupational accidents") OR ti,ab(injur* OR illness* OR claimant*) OR ti,ab(insurance NEAR/2 claim*) OR ti,ab(“job disruption")

S3

(MAINSUBJECT.EXACT("Chronic Illness") OR ti,ab(chronic NEAR/2 disease*) OR ti,ab(impairment) OR

ti,ab("heart disease") OR MAINSUBJECT.EXACT("Heart Diseases") OR MAINSUBJECT.EXACT("Mental Illness") OR ti,ab("cardiovascular disease*") OR ti,ab(stroke) OR ti,ab(TBI) OR MAINSUBJECT.EXACT("Cancer") OR ti,ab(cancer) OR ti,ab("low back pain") OR ti,ab(chronic NEAR/2 pain) OR MAINSUBJECT.EXACT("Trauma") OR ti,ab(trauma) OR MAINSUBJECT.EXACT("Anxiety") OR ti,ab(anxiety) OR MAINSUBJECT.EXACT("Depression (Psychology)") OR ti,ab(depression) OR MAINSUBJECT.EXACT("Posttraumatic Stress Disorder") OR ti,ab("post-traumatic stress") OR ti,ab("posttraumatic stress") OR ti,ab(PTSD OR PTSS) OR ti,ab(workplace* NEAR/2 violence) OR MAINSUBJECT.EXACT("Aggression") OR ti,ab(bullying) OR ti,ab(disease NEAR/2 flare*) OR MAINSUBJECT.EXACT("Fatigue") OR ti,ab(fatigue) OR MAINSUBJECT.EXACT("Arthrit

is") OR ti,ab(rheumatic NEAR/2 disease*)) AND PEER(yes)

S4

S1 AND S2

S5

S1 AND S3

S6

S4 OR S5

S7

(MAINSUBJECT.EXACT("Black Americans") OR MAINSUBJECT.EXACT("African Cultural Groups") OR MAINSUBJECT.EXACT("Asian Cultural Groups") OR MAINSUBJECT.EXACT("Ethnic Groups") OR MAINSUBJECT.EXACT("Oceanic Cultural Groups") OR MAINSUBJECT.EXACT("European Cultural Groups") OR ti,ab("African American*") OR ti,ab(Asian*) OR ti,ab(Black OR Blacks) OR ti,ab(Caucasian*) OR ti,ab(cross cultural) OR ti,ab(ethnic OR ethnicity) OR ti,ab(Hispanic*) OR ti,ab(Latina* OR Latino*) OR ti,ab("Native American*") OR ti,ab(race OR races) OR ti,ab(white OR whites) OR ti,ab(Caribbean) OR

ti,ab(Aboriginal*) OR ti,ab(Metis) OR ti,ab(Inuit) OR ti,ab(Maori*) OR ti,ab("First Nation*") OR ti,ab("First People*") OR ti,ab("American Indian*") OR ti,ab(Amerindian*) OR ti,ab(Eskimo*) OR ti,ab("Native Canadian*") OR ti,ab("Native Hawaiian*") OR ti,ab("Native people*") OR ti,ab("Native population*") OR ti,ab(tribal OR tribe) OR ti,ab("North American Native") OR ti,ab(Saami OR Sami) OR ti,ab("visible minorit*") OR ti,ab(non-white) OR (ti,ab(Indian*) NOT (ti,ab(India OR India's)))) AND PEER(yes)

S8

(MAINSUBJECT.EXACT("Racism") OR ti,ab(racis*) OR ti,ab(discrim*) OR ti,ab(bias) OR ti,ab(prejud*) OR ti,ab(hostil*) OR ti,ab(harass*) OR ti,ab("unfair treat*") OR ti,ab(oppress*) OR MAINSUBJECT.EXACT("Prejudice") OR ti,ab(cultur*) OR ti,ab(religio*) OR ti,ab(migra*) OR ti,ab(refugee*) MAINSUBJECT.EXACT("Minority Groups") OR ti,ab(immigra*) OR MAINSUBJECT.EXACT("Immigrants") OR MAINSUBJECT.EXACT("Undocumented Immigrants") OR ti,ab(racial) OR ti,ab(segregation)) AND PEER(yes)

S9

S7 OR S8

S10

ti,ab(("sick list") OR "sick leave") OR ti,ab(sick* NEAR/2 absence*) OR ti,ab("social exclusion" OR "suitable duty" OR "suitable duties" OR "suitable employment") OR ti,ab(support NEAR/2 (coworker* OR co-worker* OR colleague* OR manager* OR supervisor*)) OR ti,ab("time loss" OR "time lost") OR ti,ab(time NEAR/1 benefit*) OR ti,ab("wage replace*") OR ti,ab(work* NEAR/2 (accommodat* OR limit* OR maintenance OR participation OR re-integrat* OR reintegrat*)) OR ti,ab("work capacity" OR "work readiness" OR worklessness) OR ti,ab("disability management program*") OR MAINSUBJECT.EXACT("Case Management") OR ti,ab(("flexible work" OR "flexible working")) OR ti,ab(modifi* NEAR/2 (duty OR duties OR work)) OR ti,ab(("financial hardship" OR "financial hardships")) OR ti,ab(("financial stress" OR "financial stresses")) OR ti,ab(poverty) OR MAINSUBJECT.EXACT("Poverty") OR ti,ab(("employment opportunities" OR "employment opportunity") NEAR/2 loss*) OR ti,ab(“future earning capacity”)

S11

S6 AND S9 AND S10

S12

(S6 AND S9 AND S10) AND pd(20010101-20210430)

**Supplement 2: Methodological quality appraisal criteria and question weight**

| **Systematic review methodological quality criteria** | **Question weight** |
| --- | --- |
| Was there a clear statement of the aims of the research and was the research design appropriate to address the aims of the research? | 2 |
| Were sampling and recruitment methods (including inclusion/exclusion criteria) clearly described and similar for all participants? | 2 |
| Was recruitment (or participation) rate reported and adequate? | 2 |
| Were there important differences between those who participated and did not participate in the study with respect to key characteristics i.e., exposure(s) (including intervention(s)) and outcome(s))? | 3 |
| Were baseline characteristics described? | 2 |
| Was the length of follow-up X amount of time or greater? | 2 |
| Was the loss of follow up (attrition) less than 35%? | 2 |
| Were there important differences between those who completed the study and those who withdrew with respect to key characteristics i.e., exposure(s) (including intervention(s)) and outcomes(s)? | 3 |
| Were the instruments/methods used to assess exposure(s) valid and reliable? | 1 |
| Does the author account for the heterogeneity of their sample through an analysis that teases apart the relationship between race? | 1 |
| Were the instruments/methods used to assess the outcome(s) valid, reliable, and not prone to important sources of measurement bias? | 3 |
| Were the outcomes described at baseline and follow-up? | 3 |
| Was data collection with respect to exposure/outcome carried out equivalently for all participants? | 3 |
| Were important covariates, confounders, or baseline differences (if necessary) accounted for in the study design and/or analysis? | 2 |
| Was there a direct between group comparison? | 3 |
